# Effects of three modes of physical activity on physical fitness and hematological parameters in older people with sarcopenic obesity: A systematic review and meta-analysis

**DOI:** 10.3389/fphys.2022.917525

**Published:** 2022-08-25

**Authors:** Min Zhuang, Mengdie Jin, Tijiang Lu, Linqian Lu, Barbara E. Ainsworth, Yu Liu, Nan Chen

**Affiliations:** ^1^ Key Laboratory of Exercise and Health Sciences of Ministry of Education, Shanghai University of Sport, Shanghai, China; ^2^ Department of Rehabilitation, Xinhua Hospital Chongming Branch, Shanghai, China; ^3^ Department of Rehabilitation, Xinhua Hospital Affiliated to Shanghai Jiaotong University School of Medicine, Shanghai, China; ^4^ College of Health Solutions, Arizona State University, Phoenix, AZ, United States; ^5^ School of Kinesiology, Shanghai University of Sport, Shanghai, China

**Keywords:** exercise, body composition, muscle mass, muscle strength, physical performance, inflammation, insulin-like growth factor 1, lipids profiles

## Abstract

**Objective:** This systematic review and meta-analysis assessed the effects of three modes of physical activity (PA) (aerobic training [AT], resistance training [RT], and aerobic combined with resistance training [MT]) on body composition (body weight [BW], body mass index [BMI] and percentage of body fat [BF%]), muscle mass (skeletal muscle mass [SM], appendicular skeletal muscle mass [ASM] and appendicular skeletal muscle mass index [ASMI]), muscle strength (handgrip strength [HG] and knee extension strength [KES]), physical performance (gait speed [GS]) and hematological parameters (inflammatory markers, insulin-like growth factor 1 [IGF-1] and lipid profiles) in older people with sarcopenic obesity (SO).

**Methods:** We searched all studies for PA effects in older people with SO from six databases published from January 2010 to November 2021. Two researchers independently screened studies, extracted data according to inclusion and exclusion criteria, and assessed the quality of included studies. Pooled analyses for pre-and post- outcome measures were performed by Review Manager 5.4. We calculated a meta-analysis with a 95% confidence interval (95% CI) and the standardized mean differences (SMD).

**Results:** 12 studies were analyzed. There were 614 older people (84.9% female) with SO, aged 58.4 to 88.4 years. Compared with a no-PA control group, AT decreased BW (SMD = −0.64, 95% CI: −1.13 to −0.16, *p* = 0.009, *I* ^*2*^ = 0%) and BMI (SMD = −0.69, 95% CI: −1.18 to −0.21, *p* = 0.005, *I* ^*2*^ = 0%); RT improved BF% (SMD = −0.43, 95% CI: −0.63 to −0.22, *p* < 0.0001, *I* ^*2*^ = 38%), ASMI (SMD = 0.72, 95% CI: 0.24 to 1.21, *p* = 0.004, *I* ^*2*^ = 0%), ASM (SMD = −0.94, 95% CI: −1.46 to −0.42, *p* = 0.0004), HG (SMD = 1.06, 95% CI: 0.22 to 1.91, *p* = 0.01, *I* ^*2*^ = 90%) and KES (SMD = 1.06, 95% CI: 0.73 to 1.39, *p* < 0.00001, *I* ^*2*^ = 14%); MT improved BMI (SMD = −0.77, 95% CI: −1.26 to −0.28, *p* = 0.002, *I* ^*2*^ = 0%), BF% (SMD = −0.54, 95% CI: −0.83 to −0.25, *p* = 0.0003, *I* ^*2*^ = 0%), ASMI (SMD = 0.70, 95% CI: 0.22 to 1.19, *p* = 0.005, *I* ^*2*^ = 0%) and GS (SMD = 0.71, 95% CI: 0.23 to 1.18, *p* = 0.004, *I* ^*2*^ = 37%). PA increased IGF-1 (SMD = 0.38, 95% CI: 0.11 to 0.66, *p* = 0.006, *I* ^*2*^ = 0%), but had no effect on inflammatory markers and lipid profiles.

**Conclusion:** PA is an effective treatment to improve body composition, muscle mass, muscle strength, physical performance, and IGF-1 in older people with SO.

## Introduction

Sarcopenic obesity (SO) refers to the combination of sarcopenia and obesity. Sarcopenia is an age-related decrease of skeletal muscle mass with a decline in muscle strength and reduced physical performance (Chen et al., 2020). Obesity is a risk factor for insulin resistance, dyslipidemia, type 2 diabetes mellitus, and cardiovascular disease (Rosen and Spiegelman, 2006). People with SO are less physically active, intake more calories, and have a higher risk of diabetes or dyslipidemia than nonobese people with and without sarcopenia (Batsis and Villareal, 2018; Lim et al., 2018). A meta-analysis of SO adults showed a 24% increased risk of all-cause mortality, especially in men, compared with adults without SO (Tian and Xu, 2016). Data from World Population Prospects: the 2019 Revision (United Nations, 2019) shows that by 2050, one in six people will be 65 years or older globally, and one in four people in Europe and North America will be 65 years or older. Currently, the global prevalence of SO is 11% in nursing homes, communities, and hospitals (Gao et al., 2021). With the aging population increasing, SO may affect 100–200 million people worldwide in the next 35 years. As SO increases the risk of hospitalization, it also can increase the economic burden on individuals and nations (Janssen et al., 2004; Withrow and Alter, 2011; Lee et al., 2016; Rossi et al., 2017). Accordingly, researchers, clinicians, and policymakers should be aware of SO, its complications, and its impact on society.

Inflammatory markers play a vital role in the progression of SO (Schrager et al., 2007). Obesity causes a chronic inflammatory state, which leads to an increase in inflammatory markers, such as interleukin-6 (IL-6) and C-reactive protein (CRP) (Sáinz et al., 2010). Elevated IL-6 (>5 Pg/ml) and elevated CRP (>6.1 μg/ml) increase the risk of losing >40% muscle strength by 2 to 3 times more than people with normal IL-6 and CRP levels (Schaap et al., 2006). Upregulation of the inflammatory marker IL-6 leads to a decrease in anabolic actions of insulin-like growth factor 1 (IGF-1) (Batsis and Villareal, 2018), which weakens the maintenance and growth of skeletal muscle (Mak et al., 2011). A low level of IGF-1 also increases the risk of hyperlipidemia (García-Fernández et al., 2008), which increases the risk of cardiovascular disease (CVD) (Nelson, 2013). In people with SO, lipid levels for triglyceride (TG) and total cholesterol (TC) are higher, and high-density lipoprotein (HDL) is lower compared to people without SO (Habib et al., 2020). Increasing IGF-1, decreasing inflammatory markers, and normalizing lipid levels may improve muscle mass and strength and reduce CVD risk in people with SO (Batsis and Villareal, 2018).

At present, there is still a lack of specific pharmacological interventions for SO (Evans et al., 2021). Non-pharmacological interventions are the most commonly used method for the treatment of SO. Many studies have indicated that physical activity (PA) is one of the most effective non-pharmacological interventions for the management of SO (Theodorakopoulos et al., 2017; Hita-Contreras et al., 2018; Hsu et al., 2019). Systematic reviews and meta-analyses in older people with SO demonstrate that PA improves body composition, muscle mass, strength, and physical performance. Changes have been observed in the percentage of body fat [BF%] (Hita-Contreras et al., 2018; Hsu et al., 2019), body weight [BW] (Hsu et al., 2019), body mass index [BMI] (Hsu et al., 2019), appendicular skeletal muscle mass [ASM] (Hita-Contreras et al., 2018), handgrip strength [HG] (Theodorakopoulos et al., 2017; Hita-Contreras et al., 2018; Hsu et al., 2019) and gait speed [GS] (Theodorakopoulos et al., 2017; Hita-Contreras et al., 2018; Hsu et al., 2019). Moreover, compared with other non-pharmacological interventions (e.g., electrical acupuncture, nutritional supplements, and dietary management), older people with SO benefit from PA in reducing BF% and increasing HG and GS (Yin et al., 2020). Studies also have shown that PA in older people with SO can decrease IL-6 (Wang et al., 2019), increase IGF-1 (Chen et al., 2017; Wang et al., 2019), and improve TC and LDL lipid profiles (Park et al., 2017), reducing inflammation and the risk of CVD.

There are some limitations to meta-analysis studies on PA and SO. Firstly, inconsistent diagnostic criteria, measurement indicators, and assessment methods of SO create high heterogeneity in the meta-analysis of SO (Martínez-Amat et al., 2018; Hsu et al., 2019; Yin et al., 2020). For example, the diagnostic criteria used to diagnose sarcopenia differ among the European Working Group on Sarcopenia (EWGSOP-2010) (Liao et al., 2017), the Foundation for the National Institutes of Health (FNIH) Sarcopenia Project (Wang et al., 2019) and others (Kim et al., 2016; Vasconcelos et al., 2016; Chen et al., 2017; Huang et al., 2017; Park et al., 2017; Liao et al., 2018). The cut-off points for measurement indicators differ for ASM, HG, and GS. Diagnosis of obesity status also was inconsistent. For example, studies used different methods to measure body fat [i.e., bioelectrical impedance analysis (BIA) and dual-energy X-ray absorptiometry (DXA)]. They also based the diagnosis of obesity on different measures of body fat [e.g., BF%, BMI, visceral fat area (VFA)]. Due to different diagnostic criteria, assessment methods, and cut-off points of sarcopenia and obesity, there are differences in the identification of SO in the included articles (Kim et al., 2016; Vasconcelos et al., 2016; Chen et al., 2017; Huang et al., 2017; Liao et al., 2017; Park et al., 2017; Chiu et al., 2018; Liao et al., 2018; Wang et al., 2019; Li et al., 2020; Banitalebi et al., 2021; Lee et al., 2021). Secondly, PA mainly included aerobic training (AT), resistance training (RT), and mixed training (MT), of which the most used is aerobic combined with resistance training. There is currently a lack of studies on the effects of AT on people with SO. In people without SO, regular AT prevents loss of skeletal muscle mass and strength (Gielen et al., 2003), increases maximal oxygen uptake (VO_2_max) (Katzmarzyk et al., 2001), decreases body fat mass (Katzmarzyk et al., 2001), improves physical performance (Bull et al., 2020), and reduces the risk of CVD (Fiuza-Luces et al., 2018). The International Exercise Recommendations in Older Adults (ICFSR): Expert Consensus Guidelines 2021 (Izquierdo et al., 2021) recommends that older people perform AT 3–7 times per week at 55%–70% of heart rate reserve. In the current meta-analysis of SO, only Hsu et al. (Hsu et al., 2019) investigated the effects of AT on older people with SO. Although they indicated that older people with SO could get benefits from RT and MT, there was not enough evidence showing AT’s effects on older people with SO. Many studies have proved the benefits of AT for healthy older people or older people with chronic diseases (Chodzko-Zajko et al., 2009; Bull et al., 2020; Izquierdo et al., 2021); however, there is insufficient evidence if AT is beneficial in the progression of SO. Thirdly, IGF-1 plays a vital role in the mechanism of SO and is closely related to CVD. Currently, among the meta-analyses of SO, only Hsu et al. (Hsu et al., 2019) explored the effects of different forms of PA on hematological parameters (IL-6, CRP, TC, TG, HDL, and LDL) in older people with SO. There is no meta-analysis of PA and IGF-1. Finally, up to now, few studies have investigated the effects of different modes of PA in older people with SO. It is necessary to integrate more individual studies in a meta-analysis to explore the effects of different modes of PA on the progression of SO to provide an effective intervention for the prevention and treatment of SO.

To the best of our knowledge, this is the first meta-analysis to comprehensively explore the effects of different modes of PA on physical fitness and performance outcomes in older people with SO. This meta-analysis aims to investigate the effects of three types of PA (AT, RT, and MT) on measures of body composition, muscle mass, muscle strength, physical performance, and hematological parameters in older people with SO.

## Material and methods

### Search strategy

This meta-analysis followed the Preferred Reporting Items for Systematic Reviews and Meta-Analysis (PRISMA) guidelines (Vrabel, 2015) and is registered in the PROSPERO (CRD42022301883). We searched the following six databases: PubMed, Embase, the Cochrane Library Database, Web of Science, the China National Knowledge Infrastructure (CNKI), and Wanfang Data for studies published from January 2010 to November 2021. The Mesh terms and the synonyms were used as follows: “aging,” “aged,” “aged, 80 and over,” “cognitive aging,” “frail elderly,” “sarcopenia,” “sarcopenias,” “sarcopenic,” “muscle loss,” “muscle wasting,” “muscular atrophy,” “age-related muscle loss,” “muscle insufficiency,” “muscle depletion,” “skeletal muscle depletion,” “obesity,” “obese,” “overweight,” “sarcopenic obesity,” “exercise,” “motor activity,” “movement,” “movements,” “kinesiotherapy,” “physiotherapy,” “exercise therapy,” “training,” “physical therapy,” “physical therapy modalities,” “endurance training” and “resistance training.” All search strategies are shown in Supplementary Table S1.

### Inclusion criteria

The inclusion criteria were as follows:

1) All subjects met the definition of sarcopenic obesity (according to a working group or clinical research);
2) Aged 60 and above;
3) Without diagnosed chronic diseases, such as cardiovascular disease, metabolic disease, chronic obstructive pulmonary disease (COPD), cancer, and stroke;
4) Had at least one PA intervention group;
5) the control group did not receive any PA intervention but could receive education intervention.

### Exclusion criteria

The exclusion criteria were as follows:

1) The full text was unavailable;
2) Not in English or Chinese;
3) The studies failed to provide extractable data;
4) the intervention group received intervention combined with nutritional supplementation.

### Data extraction

According to the inclusion and exclusion criteria, two authors (MZ and MJ) independently screened the title and abstract of all studies. Then the two authors (MZ and MJ) screened the remaining full text according to inclusion and exclusion criteria. If there were disagreements about the studies, the third author (NC) participated in the discussion to resolve it. The two authors (MZ and MJ) recorded the following information in Microsoft Excel 2019: 1) the study’s first author and year of publication; 2) the characteristics of the subjects, for example, sample size, gender, and age; 3) the diagnostic criteria for sarcopenic obesity; 4) the characteristics of the intervention group, such as mode, training movement, intensity, and duration days/weeks; 4) control group intervention; 5) outcomes (e.g., body composition, muscle mass, muscle strength, physical performance, and hematological parameters). One author (MZ) was responsible for extracting the data, while the other (MJ) was responsible for checking the accuracy of the data. If the study was a multi-arm intervention, the two authors (MZ and MJ) extracted only data related to the exercise and the control group. When raw data was missing, we contacted the authors by email to request the data. We excluded the article data if the author failed to reply or would not share the raw data.

### Quality assessment

The two authors (MZ and MJ) assessed the methodological quality of each included study independently, using the Physiotherapy Evidence Database (PEDro) Scale (Maher et al., 2003), which assesses the following 11 categories: 1) eligibility criteria and source; 2) random allocation; 3) concealed allocation; 4) baseline comparability; 5) blinding of subjects; 6) blinding of therapists; 7) blinding of assessors; 8) adequate follow-up (>85%); 9) intention-to-treat analysis; 10) between-group statistical comparisons; 11) reporting of point measures and measures of variability.

If the item met the criteria, it was rated one point, and if it did not meet the criteria, it was rated 0 points. An overall score of less than four points was considered poor, and 9–10 was considered excellent. Two authors (MZ and MJ) evaluated each study independently, and if there were disagreements, the third author (YL) participated in the discussion and resolved it.

### Outcome variables

Five categories of outcome variables were extracted. Body composition was measured as body weight (BW), body mass index (BMI), and body fat percent (BF%). Muscle mass was measured as appendicular skeletal muscle mass (ASM), skeletal muscle mass (SM), and appendicular skeletal muscle mass index (ASMI). Upper and lower muscle strength was measured as handgrip (HG) and knee extension strength (KES). Physical performance was measured as gait speed (GS). Hematological parameters were measured as Interleukin-6 (IL-6), c-reactive protein (CRP), Insulin-like growth factor 1 (IGF-1), and lipids for serum total cholesterol (TC), triglycerides (TG), high-density lipoprotein (HDL), and low-density lipoprotein (LDL).

### Statistical analysis

All data included in the studies were analyzed using Review Manager (RevMan 5.4; Cochrane, Lindon, United Kingdom). We used the *I* ^*2*^ statistic to assess the heterogeneity of the outcomes of the studies. When *I* ^*2*^ < 50%, we used the fixed-effects model, and when *I* ^*2*^ > 50%, we used the random-effects model. We calculated the pooled effect sizes using the inverse variances, the 95% confidence interval (95% CI), and the standardized mean differences (SMD). A *p*-value < 0.05 was considered statistically significant.

## Results

### Study selection

**FIGURE 1:**
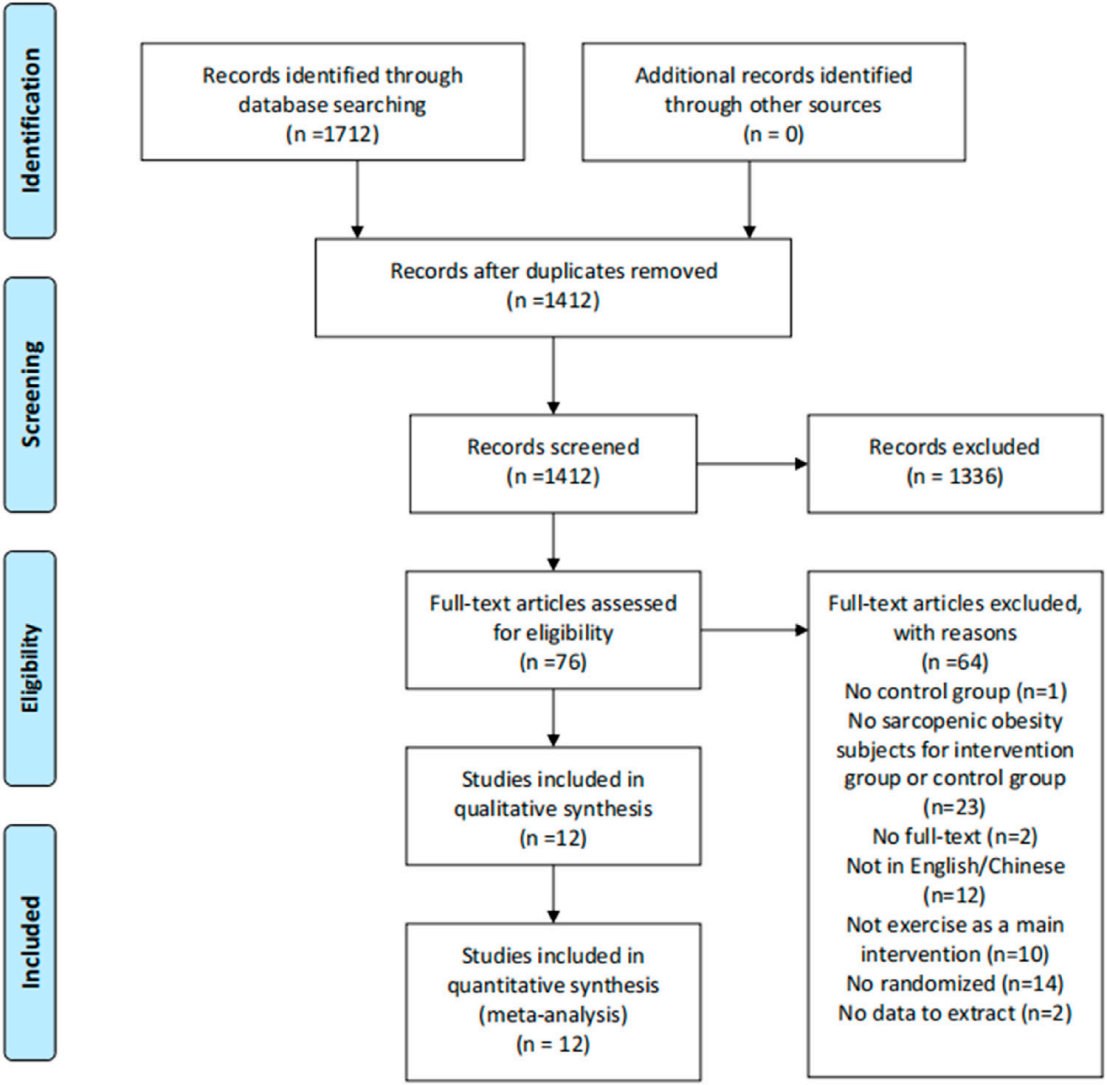
Flow of screening and selecting process according to preferred reporting items for systematic reviews and meta-analysis (PRISMA).

Figure 1 illustrates a flow chart of the study selection process and reasons for excluding studies. According to the Mesh and the synonyms published between 1 January 2010, and 30 November 2021, we identified 1712 studies from the databases. After removing 300 duplicates, 1412 studies remained. Screening the title and abstract resulted in the exclusion of 1336 studies. Of the remaining 76 studies, we excluded 64 following a full-text review according to the inclusion and exclusion criteria. The final sample included 12 studies.

### Study characteristics

Table 1 summarizes the characteristics, diagnostic criteria for SO, intervention and control group details, and the study outcomes. Subjects included 614 older people with sarcopenic obesity, aged from 58.4 to 88.4 years. Among them, 496 were female (84.93%). Eight studies (Kim et al., 2016; Vasconcelos et al., 2016; Huang et al., 2017; Liao et al., 2017; Park et al., 2017; Liao et al., 2018; Banitalebi et al., 2021; Lee et al., 2021) included women only, and none included men only. Four studies (Chen et al., 2017; Chiu et al., 2018; Wang et al., 2019; Li et al., 2020) included women and men. Diagnostic criteria for sarcopenia and obesity, the intervention and control group methods, and outcomes varied among the studies.

**TABLE 1:** Characteristics of included studies.

Study	Sample size (ETG/CG)	Gender (n: Male/female)	Age (ETG/CG)	Sarcopenia diagnostics (indicator, cut-points, Source)	Obesity diagnostics (indicator, cut-points, Source)	Intervention				Control group	Outcome
						Mode	Training movement	Intensity	Duration days/week (weeks)		
Huang et al. (2017)	18/17	0/35	68.89 ± 4.91/69.53 ± 5.09	SM/weight^2^*100% by BIA<27.6 (Janssen)	BF% by BIA>30% (Liu)	RT: Elastic band	RT: Muscle group training included shoulders, arms, lower limbs, chest, and abdomen	3 sets/10 reps	3 (12)	Education	BW, BMI, BF%, TG, HDL, LDL, TC, CRP
Vasconcelos et al. (2016)	14/14	0/28	72 ± 4.6/72 ± 3.6	HG ≤ 21 kg (Fried)	BMI≥30 kg/m^2^ (Vasconcelos)	RT: Elastic band	RT: Knee exercises, hip exercises, and mini-squats	2–3 sets/12 reps (40–60% 1RM) for knee exercises; 2–3 sets/12 reps (1–3 kg) for hip exercises; 2–3 sets/10 reps (1–3 kg) for mini-squats	2 (10)	Non-exercise	KES, GS
Liao et al. (2018)	33/23	0/56	66.67 ± 4.54/68.32 ± 6.05	SM/weight*100% by BIA<27.6% (Janssen)	BF% by BIA>30% (Liu)	RT: Elastic band	RT: Upper body exercises included seated chest press, seated row, seated shoulder press; Lower body exercises included knee extension, knee flexion, hip flexion, and hip extension	3 sets/10 reps; RPE = 13	3 (12)	Non-exercise	BF%, SM, HG, KES, GS
Liao et al. (2017)	25/21	0/46	66.39 ± 4.49/68.42 ± 5.86	SM/height^2^ by BIA<7.15 kg/m^2^ (EWGSOP-2010)	BF% by BIA>30% (Baumgartner)	RT: Elastic band	RT: Seated chest press, seated row, seated shoulder press, concentric–eccentric hip circumduction, leg press, leg curl	3 sets/10–20 repsRPE = 13	3 (12)	Non-exercise	BF%, HG, KES, GS
Chiu et al. (2018)	36/34	35/35	79.64 ± 7.36/80.15 ± 8.26	SM/weight*100% by BIA, M: ≤37.15%F: ≤32.26% (Janssen)	BF%, F: ≥29%, M: ≥40% (Ko)	RT: Sandbag and grip ball	RT: Upper extremities training that targeted the biceps, deltoids, grip, and pinch; Lower extremities training included leg extension, leg flexion, calf raises, stepping forward and sideward	3 sets/4–10 reps	2 (12)	Non-exercise	BF%, ASM, HG
Park et al. (2017)	25/25	0/50	73.5 ± 7.1/74.7 ± 5.1	ASM/weight*100% by BIA<25.1% (Lim)	BMI≥25.0 kg/m^2^ (Lim)	RT: Elastic band AT:Walking	RT: elbow flexion, wrist flexion, shoulder flexion, lateral raise, front raise, chest press, reverse flies, side band, dead lift, squat, leg press, ankle plantar flexion AT: sideways, backward, forward walking, slow and fast indoor walking	All 50–80 min, RT:2-3sets/8–15reps,20–30 min/session; AT:30–50min/session with the RPE = 13–17	RT: 3 (24), AT: 5 (24)	Education	BF%, ASM, HG, GS, TG, HDL, LDL, TC, CRP
Chen et al. (2017)	RT:15, AT:15, RT + AT:15, CG:15	10/50	RT:68.9 ± 4.4, AT:69.3 ± 3.0, RT + AT:68.5 ± 2.7, CG:68.6 ± 3.1	ASM/Weight*100%, M: ≤32.5%, F: ≤25.7% (Chung)	BMI≥ 25 kg/m^2^ (WHO); VFA≥ 100 cm^2^ (Lu)	RT: Weight-training equipment, AT: dance steps class	RT: shoulder presses, bicep curls, triceps curls, bench presses, deadlifts, leg swings, squats, standing rows, unilateral rows, and split front squats. AT: stepping on the spot, knee lifts, high knee running, rowing arm swings, arm swings, twist steps, arm raises, squats, V steps, mambo steps, diamond steps, and point step jumps	RT: 3 sets/8–12 reps. AT: moderate intensity (>3 metabolic equivalents)	RT: 2 (8), AT: 2 (8), RT + AT: 1 (8)	Non-exercise	BW, BMI, BF%, ASMI, SM, HG, KES, IGF-1
Kim et al. (2016)	RT + AT:35, CG:34	0/69	81.4 ± 4.3/81.1 ± 5.1	SM/height^2^ by DXA<5.67 kg/m^2^ or HG < 17.0 kg, or GS < 1.0 m/s (Kim-2016)	BF% by DXA≥32% (Kim-2016)	RT: Weight machines, Elastic band. AT: Stationary bicycle	RT: toe raises, heel raises, knee lifts, and knee extension, hip flexion, seated row, leg press, abduction, leg extension, and abdominal crunch. AT: Stationary bicycle	RT: 1–3/10 reps, AT: 12 min	RT:2 (12), AT:2 (12)	Education	BW, BF%, ASM, HG, KES, GS, TG, TC, IL-6, CRP
Wang et al. (2019)	RT:20, AT:20, RT + AT:20, CG:20	43/37	RT:65.1 ± 3.4, AT:64.2 ± 3.0, RT + AT:63.6 ± 5.2, CG:64.1 ± 2.8	ASM by DXA, M: <7 kg/m^2^, F:<5.4 kg/m^2^; HG, M: <26kg, F:<18 kg	BMI, M: < 0.789, F: < 0.512 (FNIH)	—	RT: Hands, feet, abdomen, pelvis and back muscle training, AT: Stepping, knee lift, leg lift, arm swing, arm lift, diamond step and dot step jump	RT:3-5sets/10–15reps, AT: 40%–60%, VO_2_max, RT + AT: RT for 10 min, AT for 20 min	RT: 2 (8), AT: 2 (8), RT + AT: 2 (8)	Non-exercise	BW, BMI, BF%, ASMI, HG, KES, IL-6, IGF-1
Li et al. (2020)	RT + AT:15, CG:15	—	RT + AT: 63.87 ± 3.56, CG:64.93±3.84	ASM/height^2^ by DXA, M: ≤7.0 kg/m^2^, F: ≤5.4 kg/m^2^ (AGWS-2013)	BF% by DXA, M: ≥25%, F: ≥35% (WHO)	RT: Elastic band, AT: Speed walking	RT: Major muscle groups training of limbs and trunk, AT: Speed walking	RT:1-3sets/10reps, RPE = 5–6/10, RPE = 5–6/10, AT: 60%–80% HRmax	RT: 3 (12), AT: 5 (12)	Non-exercise	BF%, ASM
Banitalebi et al. (2021)	RT:32, CG:31	0/63	RT:64.11± 3.81,CG:64.05± 3.35	SM/Weight^*^100% by DXA≤ 28% or SM/height^2^ by DXA ≤28% or ≤7.76 kg/m^2^; GS ≤ 1 m/s (Newman)	BF% by DXA≥32%; BMI by DXA >30 kg/m^2^ (ASBP)	RT: Elastic band	RT: major muscle groups training (legs, back, abdomen, chest, shoulders, and arms)	RT: 1–2/12 reps	RT: 3 (12)	Non-exercise	BW, BMI, BF%
Lee et al. (2021)	RT:15,CG:12	0/27	RT:70.13 ± 4.41, CG:71.82 ± 5.23	ASM/height^2^ by DXA <5.67 kg/m^2^ and HG < 20 kg or GS < 0.8 m/s (EWGSOP-2010)	BF% by DXA,>35% (Li)	RT: Elastic band	RT: major muscle groups training (shoulders, arms, lower limbs, chest, and abdomen)	RT: 3 set/10 reps	RT: 3 (12)	Non-exercise	BF%, SM, HG, GS

ETG, exercise training group; CG, control group; RT, resistance training; AT, aerobic training; reps, repetition; RPE, rated perceived exercise; 1RM, one repetition maximum; W, week; BIA, bioelectrical impedance analysis; DXA, dual energy X-ray absorptiometry; BW, body weight**;** BMI, body mass index; BF%, percentage body fat; SM, skeletal muscle mass; ASM, appendicular skeletal muscle mass; ASMI, appendicular skeletal muscle mass index; HG, handgrip strength; GS, gait speed; KES, knee extension strength; TG, triglyceride; TC, total cholesterol; HDL, high density lipoprotein; LDL, low density lipoprotein; IL-6, interleukin-6; CRP, C-reactive protein; IGF-1, insulin-like growth factor 1; HRmax, maximal heart rate; EWGSOP, European Working Group on Sarcopenia in Older People; FNIH, Foundation for the National Institutes of Health; AWGS, Asian Working Group for Sarcopenia; ASBP, the American Society of Bariatric Physicians; Age is expressed as mean ± standard deviation;

### Diagnostic criteria for sarcopenia

Table 2 shows the diagnostic criteria for sarcopenia among studies in the meta-analysis. The diagnostic criteria came from the 2010 version of the European Working Group on Sarcopenia in Older People [EWGSOP-2010 (Cruz-Jentoft et al., 2010)], the 2013 version of the Asian Working Group for Sarcopenia criteria [AGWS-2013 (Chen et al., 2014)], the Foundation for the National Institutes of Health [FNIH (Studenski et al., 2014)], and diagnostic criteria from specific research groups [Janssen (Janssen et al., 2002), Chung (Chung et al., 2013), Newman (Newman et al., 2003), Lim (Lim et al., 2010), Kim (Kim et al., 2016), and Fried (Fried et al., 2001)]. The diagnostic criteria reflect the region-specific cut-off points for muscle mass consisting of Europe [EWGSOP-2010 (Cruz-Jentoft et al., 2010)], Asia [AWGS-2013 (Chen et al., 2014)], the United States [FNIH (Studenski et al., 2014), Janssen (Janssen et al., 2002), Newman (Newman et al., 2003) and Fried (Fried et al., 2001)], Korea [Chung (Chung et al., 2013) and Lim (Lim et al., 2010)] and Japan [Kim (Kim et al., 2016)].

**TABLE 2:** Different indicators and cut-off points in defining sarcopenia.

Diagnosis criteria	Target district	Cut-off points		
		Muscle mass	Muscle strength	Muscle performance
EWGSOP-2010 Cruz-Jentoft et al. (2010)	countries from Europe	ASM/height^2^ by DXA: (M:<7.26 kg/m^2^, F:<5.50 kg/m^2^); or SM/height^2^ by BIA: (M:≤8.87 kg/m^2^, F:≤6.42 kg/m^2^)	HG: (M:<30 kg, F:<20 kg)	GS (4 m): <0.8 m/s; or GS (6 m): < 1 m/s, or SPPB: ≤ 8
AWGS-2013 Chen et al. (2014)	countries from Asia	ASM/height^2^ by DXA: (M:≤7.0 kg/m^2^, F:≤5.4 kg/m^2^); or ASM/height^2^ by BIA: (M: ≤7.0 kg/m^2^, F: ≤5.7 kg/m^2^)	HG: (M:<26 kg, F:<18 kg)	GS (6 m): <0.8 m/s
FNIH Studenski et al. (2014)	United States	ASM/BMI by DXA: (M < 0.789, F < 0.512)	HG: (M < 26kg, F < 16 kg)	—
Janssen et al. (2002)	United States	[(height^2^/BIA-resistance * 0.401) + 3.825 (gender)+ 0.071 (age)+ 5.102]/body mass * 100] <1 standard deviations of a young reference population	—	—
Chung et al. (2013)	Korea	ASM/weight * 100% by DXA, M: ≤32.5%, F: ≤25.7%	—	—
Newman et al. (2003)	United States	F:ALM (kg) = −13.19 + 14.75*height (m) + 0.23 * total fat mass (kg). M:ALM (kg) = −22.48 + 24.14 * height(m) + 0.21 * total fat mass (kg),the 20th percentile of the distribution of residuals	—	—
Lim et al. (2010)	Korea	ASM/height^2^ by DXA: (M < 7.09 kg/m^2^,F < 5.27 kg/m^2^); or ASM/weight * 100% by DXA: (M<29.9%, F<25.1%)	—	—
Kim et al. (2016)	Japan	SM/height^2^ by DXA <5.67 kg/m^2^	HG: <17.0 kg	GS (5 m): <1.0 m/s
Fried et al. (2001)	United States	weight loss>10 poundsor ≥5% of body weight of the previous year	HG: lowest 20% (by gender, BMI)	GS: slowest 20% (by gender, height)

BIA: bioelectrical impedance analysis; DXA: dual energy X-ray absorptiometry; BMI: body mass index; SM: skeletal muscle mass (kg); ASM: appendicular skeletal muscle mass; ALM: appendicular lean mass; HG: handgrip strength; GS: gait speed; SPPB: the short physical performance battery; TUG: time up and go test; EWGSOP: European Working Group on Sarcopenia in Older People; FNIH: Foundation for the National Institutes of Health; AWGS: Asian Working Group for Sarcopenia; M: male; F: female.

The studies used various criteria to measure sarcopenia. Two studies used EWGSOP-2010 (Liao et al., 2017; Lee et al., 2021), one study used AGWS-2013 (Li et al., 2020), three studies used Janssen (Huang et al., 2017; Chiu et al., 2018; Liao et al., 2018), and one study each used criteria from FNIH (Wang et al., 2019), Chung (Chen et al., 2017), Newman (Banitalebi et al., 2021), Lim (Park et al., 2017), Kim (Kim et al., 2016), and Fried (Vasconcelos et al., 2016).

### Diagnostic criteria for obesity

Table 3 shows the methods and cut-off points used to classify obesity, including the names of people and organizations creating the cut-off points to classify obesity in international locations. The methods included BF% measured by bioelectrical impedance analysis (BIA) and Dual Energy X-ray Absorptiometry (DXA), visceral fat area (VFA) measured by computed tomography (CT), and BMI calculated as weight kilogram/height meter^2^.

**TABLE 3:** Different indicators and cut-off points in defining obesity.

Diagnosis criteria	Target district	Cut-off points
Deurenberg et al. (1998)	America, Caucasia, China, Ethiopia, Indonesia, Polynesia and Thailand	BF% by BIA>30%
Ko et al. (2001)	China	BF% by BIA,F: ≥29%,M: ≥40%
Vasconcelos et al. (2016)	Brazil	BMI≥30 kg/m^2^
Baumgartner. (2000)	New Mexico	BF% by BIA>30%
Lim et al. (2010)	Korea	VFA by abdominal CT > 100 cm^2^
FNIH Studenski et al. (2014)	—	BMI, M:<0.789, F:< 0.512
Kim et al. (2016)	Japan	BF% by DXA, ≥ 32%
WHO Use and Anthropometry. (1995)	Asia	BF% by DXA, M: ≥ 25%, F: ≥ 35%; BMI ≥25 kg/m^2^
Li et al. (2012)	China	BF% by BIA, M: ≥ 25%, F: ≥ 35%
ASBP Ilich et al. (2016)	United States	BF% by DXA ≥32%

BIA, bioelectrical impedance analysis; DXA, dual energy X-ray absorptiometry; BF%, body fat percentage; VFA, visceral fat area; CT, computed tomography; BMI, body mass index; M, male; F, female; FNIH, foundation for the national institutes of health; WHO, world health organization; ASBP, american society of bariatric physicians.

Seven studies measured obesity with BF% (Kim et al., 2016; Huang et al., 2017; Liao et al., 2017; Chiu et al., 2018; Liao et al., 2018; Li et al., 2020; Lee et al., 2021) three studies used BMI (Vasconcelos et al., 2016; Park et al., 2017; Wang et al., 2019), one study used BMI and VFA (Chen et al., 2017), and one study used BF% and BMI (Banitalebi et al., 2021). No studies only used VFA. Sources for the diagnostic criteria for obesity used in studies were Deurenberg (Huang et al., 2017; Liao et al., 2018), Ko (Chiu et al., 2018), Vasconcelos (Vasconcelos et al., 2016), Baumgartner (Liao et al., 2017), Lim (Park et al., 2017), World Health Organization (WHO) (Chen et al., 2017; Li et al., 2020), FNIH (Wang et al., 2019), Kim (Kim et al., 2016), American Society of Bariatric Physicians (ASBP) (Banitalebi et al., 2021) and Li (Lee et al., 2021). The diagnostic criteria for obesity vary, and the applications and cut-off points used to classify obesity may be regional. In general, most studies use the cut-off points for obesity of BF% ≥ 25% and BMI ≥30 kg/m^2^.

### Quality assessment

Table 4 shows the PEDro scores of the 12 studies. One was rated 10, considered “excellent.” Nine were rated six to eight, considered “good.” Two were rated 5, considered “fair.” All studies met eligibility criteria for group comparison, point measures reporting, and measures of variability. Eleven studies were allocated randomly. Nine studies had concealed allocation. The baselines were similar for the 12 studies. Ten studies had adequate follow-up (>85%). Two studies blinded both participants and therapists. Seven studies blinded the assessors.

**TABLE 4:** PEDro criteria and scores of included studies.

Study	Eligibility criteria	Random allocation	Concealed allocation	Baseline similar	Blinding (subject)	Blinding (therapists)	Blinding (assessor)	Measure for>85%	Intention-to-treat analysis	Group comparison	Point measures	Total score (0–10)
Huang et al. (2017)	yes	1	1	1	0	0	1	1	1	1	1	8
Vasconcelos et al. (2016)	yes	1	1	1	0	0	1	1	1	1	1	8
Liao et al. (2018)	yes	1	1	1	0	0	1	1	1	1	1	8
Liao et al. (2017)	yes	1	1	1	1	1	1	1	1	1	1	10
Chiu et al. (2018)	yes	0	0	1	1	1	0	0	0	1	1	5
Park et al. (2017)	yes	1	0	1	0	0	1	1	1	1	1	7
Chen et al. (2017)	yes	1	0	1	0	0	1	0	0	1	1	5
Kim et al. (2016)	yes	1	1	1	0	0	0	1	0	1	1	6
Wang et al. (2019)	yes	1	1	1	0	0	0	1	0	1	1	6
Li et al. (2020)	yes	1	1	1	0	0	0	1	1	1	1	7
Banitalebi et al. (2021)	yes	1	1	1	0	0	0	1	1	1	1	7
Lee et al. (2021)	yes	1	1	1	0	0	1	1	1	1	1	8

PEDro, Physiotherapy Evidence Database; 1, meet the standard; 0, not meet the standard.

### Outcomes

#### Effects of different exercise modes for sarcopenic obesity on body composition

**FIGURE 2:**
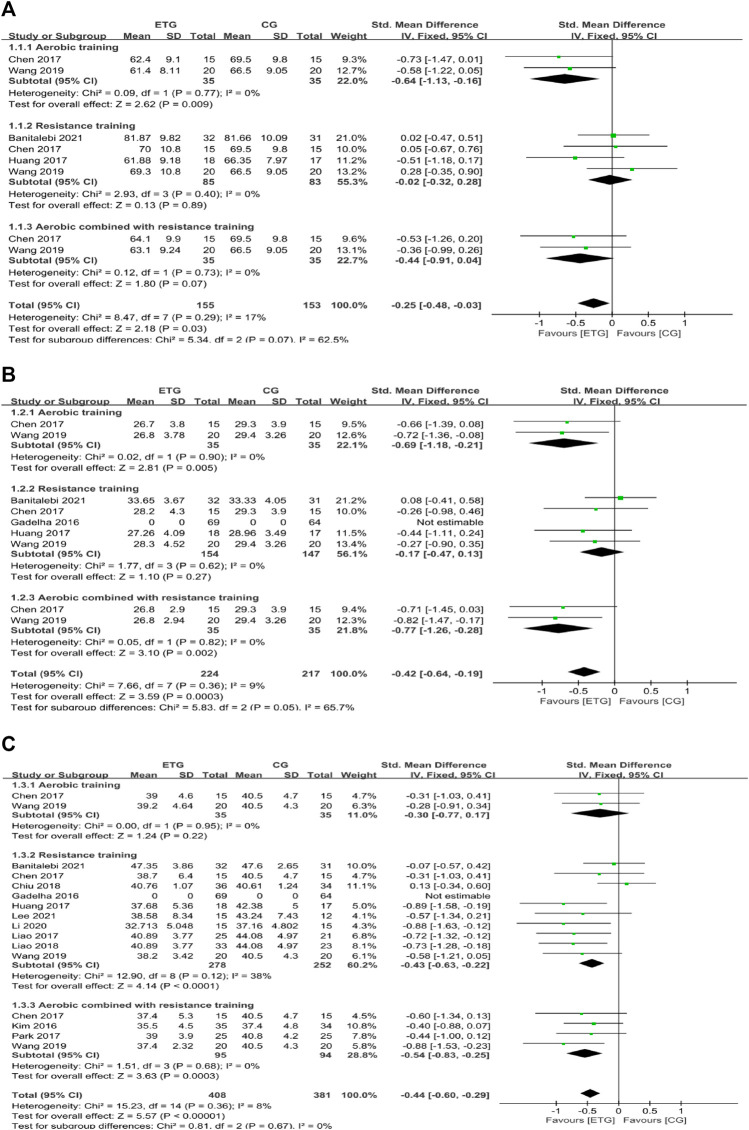
Forest plots of the comparison of the exercise training group (ETG) versus the control group (CG) on **(A)** body weight (BW); **(B)** body mass index (BMI); **(C)** percentage of body fat (BF%); CI: confidence interval; SD: standard deviation.

Eleven of the 12 studies assessed the effects of different exercise modes on body composition (BW, BMI, and BF%). Four explored the effects of different exercise modes on BW (Chen et al., 2017; Huang et al., 2017; Wang et al., 2019; Banitalebi et al., 2021) (Figure 2). Two studies included all three exercise modes (Chen et al., 2017; Wang et al., 2019). AT improved BW compared with the control group (SMD = −0.64, 95% CI: −1.13 to −0.16, *p* = 0.009, *I* ^*2*^ = 0%) (Chen et al., 2017; Wang et al., 2019). RT showed no significant difference in BW compared with the control group (SMD = −0.02, 95% CI: −0.32 to 0.28, *p* = 0.89, *I* ^2^ = 0%) (Chen et al., 2017; Huang et al., 2017; Wang et al., 2019; Banitalebi et al., 2021). MT showed no significant difference in BW compared with the control group (SMD = −0.44, 95% CI: −0.91 to 0.04, *p* = 0.07, *I* ^*2*^ = 0%) (Chen et al., 2017; Wang et al., 2019). Collectively, the three exercise modes showed a significant decrease in BW compared with the control group (SMD = −0.25, 95% CI: −0.48 to −0.03, *p* = 0.03, *I* ^*2*^ = 17%).

Four of the 12 studies explored the effects of different exercise modes on BMI (Chen et al., 2017; Huang et al., 2017; Wang et al., 2019; Banitalebi et al., 2021). Two studies included all three exercise modes (Chen et al., 2017; Wang et al., 2019). AT improved BMI compared with the control group (SMD = −0.69, 95% CI: −1.18 to −0.21, *p* = 0.005, *I* ^*2*^ = 0%) (Chen et al., 2017; Wang et al., 2019). RT showed no significant difference in BMI compared with the control group (SMD = −0.17, 95% CI: −0.47 to 0.13, *p* = 0.27, *I* ^*2*^ = 0%) (Chen et al., 2017; Huang et al., 2017; Wang et al., 2019; Banitalebi et al., 2021). MT improved BMI compared with the control group (SMD = −0.77, 95% CI: −1.26 to −0.28, *p* = 0.002, *I* ^*2*^ = 0%) (Chen et al., 2017; Wang et al., 2019). Collectively, all exercise modes showed a significant decrease in BMI compared with the control group (SMD = −0.42, 95% CI: −0.64 to −0.19, *p* = 0.0003, *I* ^*2*^ = 9%).

Eleven of the 12 studies explored the effects of different exercise modes on BF% (Kim et al., 2016; Chen et al., 2017; Huang et al., 2017; Liao et al., 2017; Park et al., 2017; Chiu et al., 2018; Liao et al., 2018; Wang et al., 2019; Li et al., 2020; Banitalebi et al., 2021; Lee et al., 2021). Two studies included all three exercise modes (Chen et al., 2017; Wang et al., 2019). AT showed no significant difference in BF% compared with the control group (SMD = −0.30, 95% CI: −0.77 to 0.17, *p* = 0.22, *I* ^*2*^ = 0%) (Chen et al., 2017; Wang et al., 2019). RT improved BF% compared with the control group (SMD = −0.43, 95% CI: −0.63 to −0.22, *p* < 0.0001, *I* ^*2*^ = 38%) (Chen et al., 2017; Huang et al., 2017; Liao et al., 2017; Chiu et al., 2018; Liao et al., 2018; Wang et al., 2019; Li et al., 2020; Banitalebi et al., 2021; Lee et al., 2021). MT improved BF% compared with the control group (SMD = −0.54, 95% CI: −0.83 to −0.25, *p* = 0.0003, *I* ^*2*^ = 0%) (Kim et al., 2016; Chen et al., 2017; Park et al., 2017; Wang et al., 2019). Collectively, different exercise modes showed a significant decrease in BF% compared with the control group (SMD = −0.44, 95% CI: −0.60 to −0.29, *p* < 0.00001, *I* ^*2*^ = 8%).

#### Effects of different exercise modes on muscle mass

There were three outcomes of muscle mass: SM, ASM, and ASMI.

**FIGURE 3:**
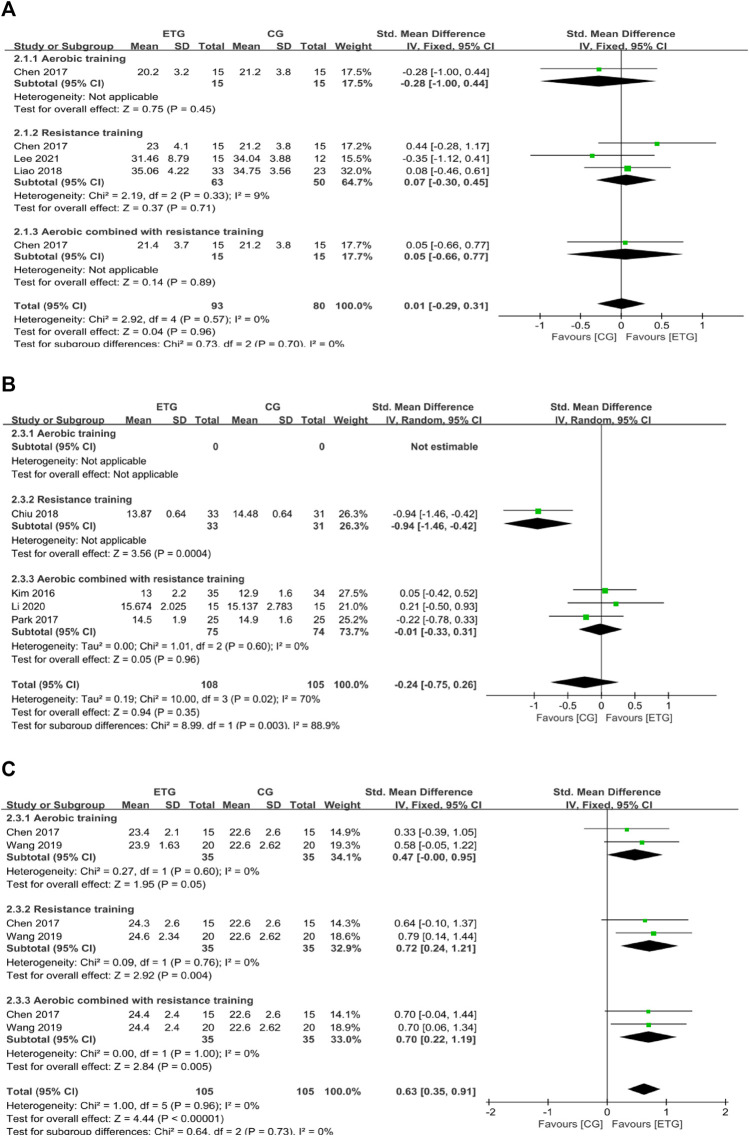
Forest plots of the comparison of the exercise training group (ETG) versus the control group (CG) on **(A)** skeletal muscle mass (SM); **(B)** appendicular skeletal muscle mass (ASM); and **(C)** appendicular skeletal muscle mass index (ASMI); CI, confidence interval; SD, standard deviation.

Three of the 12 studies explored the effects of different exercise modes on SM (Chen et al., 2017; Liao et al., 2018; Lee et al., 2021) (Figure 3). One study included all three exercise modes (Chen et al., 2017) and two studies included only RT (Liao et al., 2018; Lee et al., 2021). Results showed no significant difference between exercise groups and control group (AT, SMD = −0.28, 95% CI: −1.00 to 0.44, *p* = 0.45; RT, SMD = 0.07, 95% CI: −0.30 to 0.45, *p* = 0.71, *I* ^*2*^ = 9%; MT, SMD = 0.05, 95% CI: −0.66 to 0.77, *p* = 0.89). Collectively, different exercise modes showed no significant difference in SM compared with the control group (SMD = 0.01, 95% CI: −0.29 to 0.31, *p* = 0.96, *I* ^*2*^ = 0%).

Four of the 12 studies explored the effects of different exercise modes on ASM (Kim et al., 2016; Park et al., 2017; Chiu et al., 2018; Li et al., 2020). RT improved ASM compared with the control group (SMD = −0.94, 95% CI: −1.46 to −0.42, *p* = 0.0004) (Chiu et al., 2018). MT showed no significant differences between exercise modes with the control groups (SMD = −0.01, 95% CI: −0.33 to 0.31, *p* = 0.96, *I* ^*2*^ = 0%) (Kim et al., 2016; Park et al., 2017; Li et al., 2020). Collectively, the different exercise modes showed no significant difference in ASM compared with the control group (SMD = −0.24, 95% CI: −0.75 to 0.26, *p* = 0.35, *I* ^*2*^ = 70%).

Two of the 12 studies explored the effects of different exercise modes on ASMI (Chen et al., 2017; Wang et al., 2019). Two studies included all three exercise modes (Chen et al., 2017; Wang et al., 2019). AT showed no significant difference in ASMI compared with the control group (SMD = 0.47, 95% CI: −0.00 to 0.95, *p* = 0.05, *I* ^*2*^ = 0%) (Chen et al., 2017; Wang et al., 2019). RT showed a significant increase in ASMI compared with the control group (SMD = 0.72, 95% CI: 0.24 to 1.21, *p* = 0.004, *I* ^*2*^ = 0%) (Chen et al., 2017; Wang et al., 2019). MT showed a significant increase in ASMI compared with the control group (SMD = 0.70, 95% CI: 0.22 to 1.19, *p* = 0.005, *I* ^*2*^ = 0%) (Chen et al., 2017; Wang et al., 2019). Collectively, different exercise modes showed a significant increase in ASMI compared with the control group (SMD = 0.63, 95% CI: 0.35 to 0.91, *p* < 0.00001, *I* ^*2*^ = 0%).

#### Effects of different exercise modes on muscle strength

**FIGURE 4:**
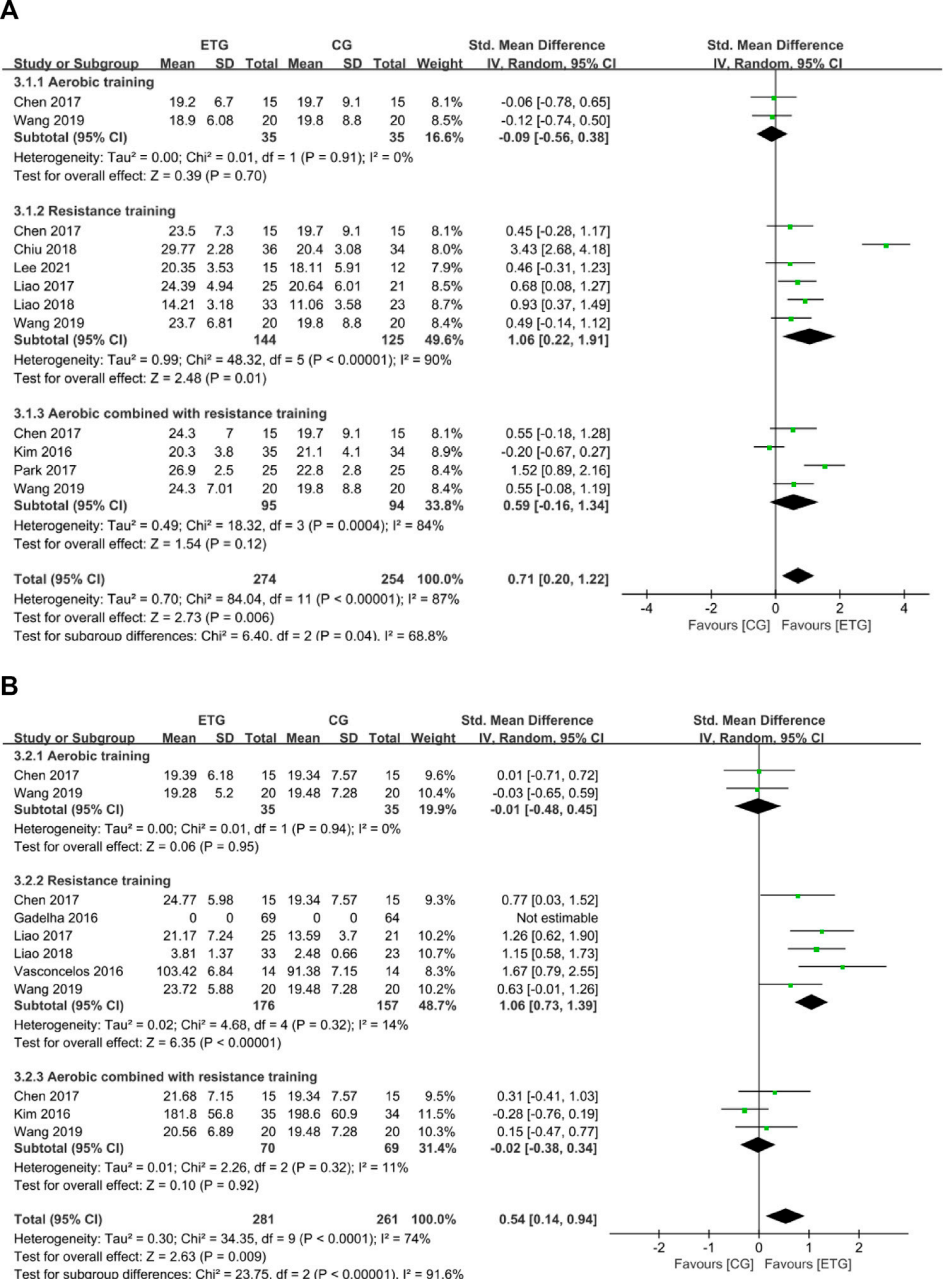
Forest plots of the comparison of the exercise training group (ETG) versus the control group (CG) on **(A)** handgrip strength (HG); **(B)** knee extension strength (KES); CI, confidence interval; SD, standard deviation.

There were two outcomes of muscle strength: HG and KES. Eight of the 12 studies explored the effects of different exercise modes on HG (Kim et al., 2016; Chen et al., 2017; Liao et al., 2017; Park et al., 2017; Chiu et al., 2018; Liao et al., 2018; Wang et al., 2019; Lee et al., 2021) (Figure 4). AT showed no significant difference in HG compared with the control group (SMD = −0.09, 95% CI: −0.56 to 0.38, *p* = 0.70, *I* ^*2*^ = 0%) (Chen et al., 2017; Wang et al., 2019). RT showed a significant increase in HG compared with the control group (SMD = 1.06, 95% CI: 0.22 to 1.91, *p* = 0.01, *I* ^*2*^ = 90%) (Chen et al., 2017; Liao et al., 2017; Chiu et al., 2018; Liao et al., 2018; Wang et al., 2019; Lee et al., 2021). MT showed no significant difference in HG compared with the control group (SMD = 0.59, 95% CI: -0.16 to 1.34, *p* = 0.12, *I* ^*2*^ = 84%) (Kim et al., 2016; Chen et al., 2017; Park et al., 2017; Wang et al., 2019). Collectively, different exercise modes showed a significant increase in HG compared with the control group (SMD = 0.71, 95% CI: 0.20 to 1.22, *p* = 0.006, *I* ^*2*^ = 87%).

Six of the twelve studies explored the effects of different exercise modes on KES (Kim et al., 2016; Vasconcelos et al., 2016; Chen et al., 2017; Liao et al., 2017; Liao et al., 2018; Wang et al., 2019). Two studies included all three exercise modes (Chen et al., 2017; Wang et al., 2019). AT showed no significant difference in KES compared with the control group (SMD = -0.01, 95% CI: −0.48 to 0.45, *p* = 0.95, *I* ^*2*^ = 0%) (Chen et al., 2017; Wang et al., 2019). RT showed a significant increase in KES compared with the control group (SMD = 1.06, 95% CI: 0.73 to 1.39, *p* < 0.00001, *I* ^*2*^ = 14%) (Vasconcelos et al., 2016; Chen et al., 2017; Liao et al., 2017; Liao et al., 2018; Wang et al., 2019). MT showed no significant difference in KES compared with the control group (SMD = -0.02, 95% CI: −0.38 to 0.34, *p* = 0.92, *I* ^*2*^ = 11%) (Kim et al., 2016; Chen et al., 2017; Wang et al., 2019). Collectively, different exercise modes showed a significant increase in KES compared with the control group (SMD = 0.54, 95% CI: 0.14 to 0.94, *p* = 0.009, *I* ^*2*^ = 74%).

#### Effects of different exercise modes for sarcopenic obesity on the physical performance

**FIGURE 5:**
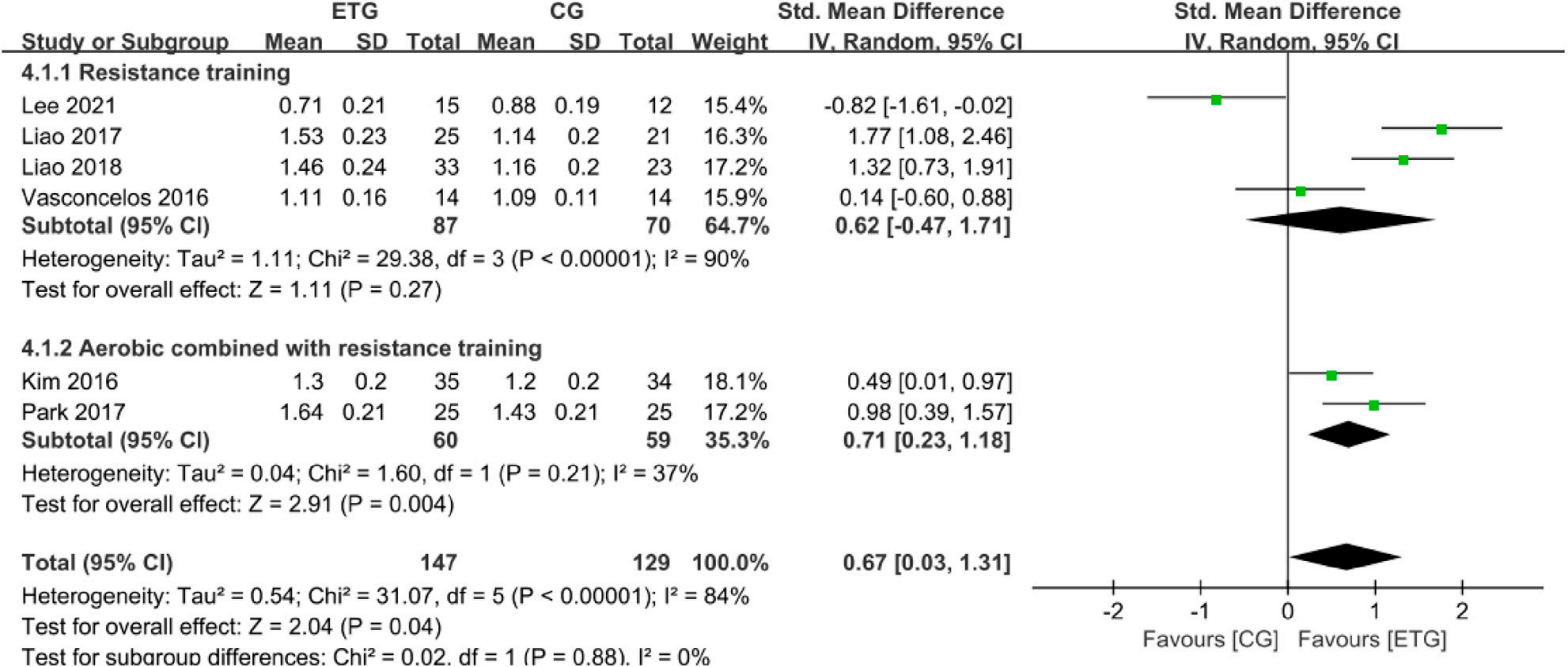
Forest plots of the comparison of the exercise training group (ETG) versus the control group (CG) on gait speed (GS); CI, confidence interval; SD, standard deviation.

Six of the 12 studies explored the effects of different exercise modes on GS (Kim et al., 2016; Vasconcelos et al., 2016; Liao et al., 2017; Park et al., 2017; Liao et al., 2018; Lee et al., 2021) (Figure 5). No studies explored the effect of AT on GS. RT showed no significant difference in GS compared with the control group (SMD = 0.62, 95% CI: −0.47 to 1.71, *p* = 0.27, *I* ^*2*^ = 90%) (Vasconcelos et al., 2016; Liao et al., 2017; Liao et al., 2018; Lee et al., 2021). MT showed a significant increase in GS compared with the control group (SMD = 0.71, 95% CI: 0.23 to 1.18, *p* = 0.004, *I* ^*2*^ = 37%) (Kim et al., 2016; Park et al., 2017). Collectively, different exercise modes showed a significant increase in GS compared with the control group (SMD = 0.67, 95% CI: 0.03 to 1.31, *p* = 0.04, *I* ^*2*^ = 84%).

#### Effects of different exercise modes on hematological parameters

**FIGURE 6:**
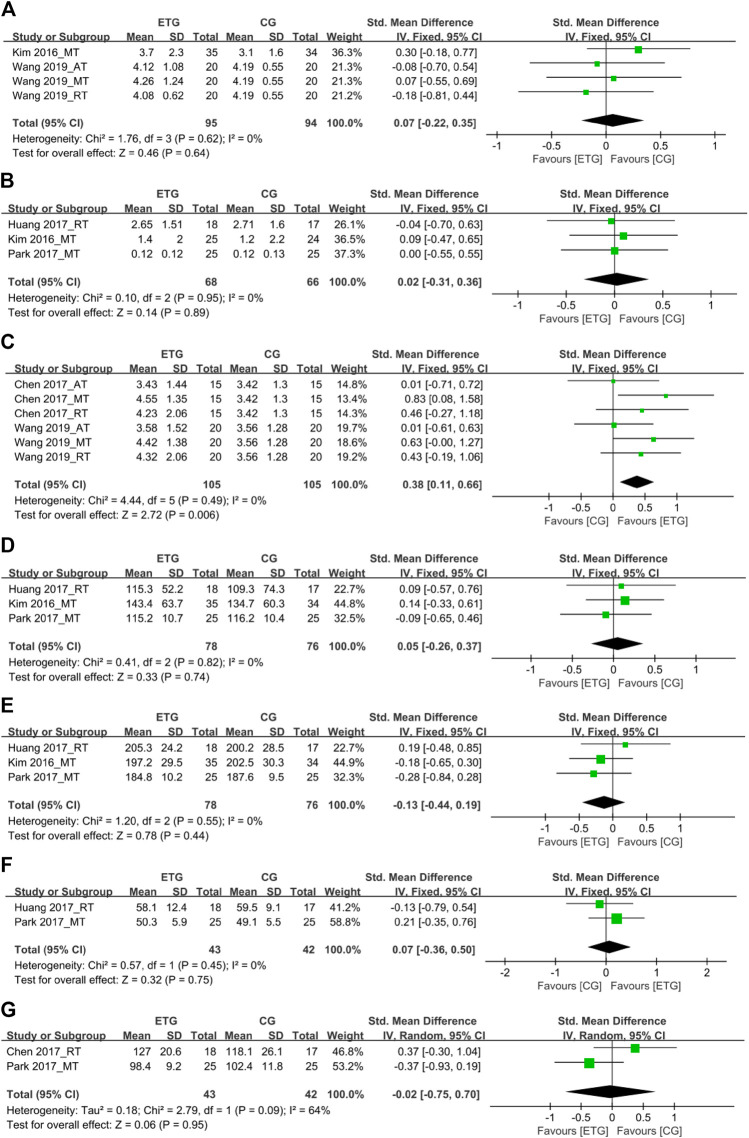
Forest plots of the comparison of the exercise training group (ETG) versus the control group (CG) on **(A)** interleukin-6 (IL-6); **(B)** C-reactive protein (CRP); **(C)** insulin-like growth factor 1 (IGF-1); **(D)** triglyceride (TG); **(E)** total cholesterol (TC); **(F)** high-density lipoprotein (HDL); **(G)** low-density lipoprotein (LDL); AT, aerobic training; RT, resistance training; MT, aerobic combined with resistance training; CI, confidence interval; SD, standard deviation.

Seven outcomes of hematological parameters included inflammatory markers (IL-6 and CRP), IGF-1, and lipid profile measures (TG, TC, HDL, and LDL). For the inflammatory markers, two of the studies explored the effects of different exercise modes on IL-6 (Kim et al., 2016; Wang et al., 2019) (Figure 6). One study included all three exercise modes (Wang et al., 2019). The other study explored the effect of MT on IL-6 (Kim et al., 2016). None of the exercise modes showed a significant difference in IL-6 compared with the control group (SMD = 0.07, 95% CI: −0.22 to 0.35, *p* = 0.64, *I* ^*2*^ = 0%). Three studies explored the effects of different exercise modes on CRP (Kim et al., 2016; Huang et al., 2017; Park et al., 2017). One study explored the effect of RT on CRP (Huang et al., 2017). Two studies explored the effect of MT on CRP (Kim et al., 2016; Park et al., 2017). No studies explored the effect of AT on CRP. None of the exercise modes showed a significant difference in CRP compared with the control group (SMD = 0.02, 95% CI: -0.31 to 0.36, *p* = 0.89, *I* ^*2*^ = 0%).

Two studies explored the effects of different exercise modes on IGF-1 (Chen et al., 2017; Wang et al., 2019). Both studies included all three exercise modes (Chen et al., 2017; Wang et al., 2019). In total, all three exercise modes showed a significant increase in IGF-1 compared with the control group (SMD = 0.38, 95% CI: 0.11 to 0.66, *p* = 0.006, *I* ^*2*^ = 0%).

Among the lipid profile markers, three studies explored the effects of RT and MT on TG (Kim et al., 2016; Huang et al., 2017; Park et al., 2017). One of the three studies explored the RT on TG (Huang et al., 2017). The other two studies explored the effect of MT on TG (Kim et al., 2016; Park et al., 2017). No studies explored the effect of AT on TG. None of the two exercise modes showed a significant difference in TG compared with the control group (SMD = 0.05, 95% CI: −0.26 to 0.37, *p* = 0.74, *I* ^*2*^ = 0%). Three studies explored the effects of different exercise modes for SO on TC (Kim et al., 2016; Huang et al., 2017; Park et al., 2017). One of the three studies explored the RT on TC (Huang et al., 2017). The other two studies explored the effect of MT on TC (Kim et al., 2016; Park et al., 2017). No studies explored the effect of AT on TC. None of the two exercise modes showed a significant difference in TC compared with the control group (SMD = −0.13, 95% CI: −0.44 to 0.19, *p* = 0.44, *I* ^*2*^ = 0%).

Two studies explored the effects of RT and MT for SO on HDL, respectively (Huang et al., 2017; Park et al., 2017). None of the exercise modes showed a significant difference in HDL compared with the control group (SMD = 0.07, 95% CI: −0.36 to 0.50, *p* = 0.75, *I* ^*2*^ = 0%). Two studies explored the effects of RT and MT for SO on LDL, respectively (Chen et al., 2017; Park et al., 2017). None of the exercise modes showed a significant difference in LDL compared with the control group (SMD = −0.02, 95% CI: −0.75 to 0.70, *p* = 0.95, *I* ^*2*^ = 64%).

### Moderator variables

To explore the influence of different exercise modes on muscle mass, inflammatory markers, and lipid profiles, we performed subgroup analyses to assess the potential effects of different moderators on the outcomes. Table 5 illustrates the influence of moderator variables on muscle mass, inflammatory markers, and lipid profiles. The subgroup analysis included the moderator variables of sarcopenia and obesity diagnostic indicators (e.g., EWGSOP-2010, BF%), intervention duration, and intervention frequency.

**TABLE 5:** Influence of moderator variables in the effect of physical activity on inflammatory markers, lipid profiles, BMD and muscle mass.

Variable	Subgroup	Studies	n	Effect size with 95% confidence interval	Heterogeneity			Test overall effects. Z(p)		Test for subgroup Difference.Chi^2^(p)	
					Chi^2^	P	I^2^				
**Inflammatory markers (IL--6 and CRP)**											
Age (years)	<70	4	155	−0.06 [−0.37, 0.26]	0.33	0.95	0	0.36 (0.72)		0.86 (0.35)	
	≥70	3	188	0.14 [−0.14, 0.43]	0.71	0.70	0	0.98 (0.33)			
Intervention duration (weeks)	<12	3	120	−0.06 [−0.42, 0.29]	0.33	0.85	0	0.35 (0.73)		0.62 (0.43)	
	≥12	4	223	0.11 [−0.15, 0.38]	0.95	0.81	0	0.86 (0.39)			
Frequency (days/week)	<3	5	258	0.07 [−0.17, 0.32]	1.77	0.78	0	0.60 (0.55)		0.13 (0.72)	
	≥3	2	85	−0.02 [−0.44, 0.41]	0.01	0.93	0	0.07 (0.94)			
Sarcopenia Assessment method	BIA	2	85	−0.02 [−0.44, 0.41]	0.01	0.93	0	0.07 (0.94)		0.13 (0.72)	
	DXA	5	258	0.07 [−0.17, 0.32]	1.77	0.78	0	0.60 (0.55)			
Sarcopenia diagnostic indicator	SM	3	173	0.15 [−0.15, 0.45]	0.74	0.69	0	0.97 (0.33)		0.80 (0.37)	
	ASM	4	170	−0.05 [−0.35, 0.26]	0.37	0.95	0	0.29 (0.77)			
Obesity diagnostic indicator	BF%	3	173	0.15 [−0.15, 0.45]	0.74	0.69	0	0.97 (0.33)		0.80 (0.37)	
	BMI	4	170	−0.05 [−0.35, 0.26]	0.37	0.95	0	0.29 (0.77)			
Lipid profiles (TC, TG, HDL LDL)									
Age (years)	<70	4	140	0.13 [−0.20, 0.46]	1.11	0.77	0	0.77 (0.44)		1.16 (0.28)	
	≥70	6	338	−0.09 [−0.30, 0.13]	3.54	0.62	0	0.79 (0.43)			
Sarcopenia assessment method	BIA	8	340	−0.02 [−0.24, 0.19]	4.96	0.66	0	0.23 (0.82)		0.00 (0.97)	
	DXA	2	138	−0.02 [−0.35, 0.32]	0.85	0.36	0	0.11 (0.91)			
Sarcopenia diagnostic indicator	SM	6	278	0.06 [−0.18, 0.29]	2.34	0.80	0	0.47 (0.64)		1.04 (0.31)	
	ASM	4	200	−0.13 [−0.41, 0.14]	2.42	0.49	0	0.94 (0.35)			
Obesity diagnostic indicator	BF%	5	243	0.08 [−0.17, 0.34]	2.01	0.73	0	0.64 (0.52)		2.51 (0.11)	
	BMI	3	150	−0.25 [−0.57, 0.07]	0.50	0.78	0	1.51 (0.13)			
Intervention duration (weeks)	≤12	6	278	0.06 [−0.18, 0.29]	2.34	0.80	0	0.47 (0.64)		1.04 (0.31)	
	>12	4	200	−0.13 [−0.41, 0.14]	2.42	0.49	0	0.94 (0.35)			
Frequency (days/week)	≤3	6	278	0.06 [−0.18, 0.29]	2.34	0.80	0	0.47 (0.64)		1.04 (0.31)	
	>3	4	200	−0.13 [−0.41, 0.14]	2.42	0.49	0	0.94 (0.35)			
BMD											
T-score	>-1 SD	12	530	−0.52 [−0.70, −0.35]	13.81	0.24	20	5.84 (<0.00001)		0.51 (0.48)
	≤-1 SD	3	119	−0.37 [−0.74, -0.01]	3.35	0.19	40	2.00 (0.05)			
**Variable**	**Subgroup**	**Studies**	n	**Effect size with 95% confidence interval**	**Heterogeneity**				**Test overall effects Z(p)**		**Test for subgroup Difference.Chi^2^(p)**
					**Tau^2^**	**Chi^2^**	**P**	**I^2^**			
SM, ASM and ASMI									
Age (years)	<65	3	110	0.52 [0.14, 0.90]	0.00	1.04	0.59	0	2.68 (0.007)		3.15 (0.08)
	≥65	12	486	0.08 [−0.22, 0.38]	0.17	28.96	0.002	62	0.54 (0.59)		
Sarcopenia Assessment method	BIA	13	539	0.20 [−0.09, 0.49]	0.18	33.50	0.0008	64	1.33 (0.18)		0.62 (0.43)
	DXA	2	57	−0.05 [−0.61, 0.50]	0.02	1.13	0.29	11	0.19 (0.85)		
Obesity diagnostic indicator	BF%	5	246	−0.20 [−0.64, 0.24]	0.16	11.42	0.02	65	0.88 (0.38)		4.67 (0.03)
	BMI	10	350	0.36 [0.11, 0.62]	0.05	12.64	0.18	29	2.80 (0.005)		
BMI levels	<27 kg/m^2^	10	390	0.09 [−0.26, 0.44]	0.21	26.36	0.002	66	0.51 (0.61)		0.64 (0.42)
	≥27 kg/m^2^	5	206	0.30 [−0.08, 0.68]	0.08	7.19	0.13	44	1.57 (0.12)		
Intervention duration (weeks)	<12	9	300	0.46 [0.23, 0.69]	0.00	7.72	0.46	0	3.87 (0.0001)		9.28 (0.002)
	≥12	6	296	−0.20 [−0.56, 0.15]	0.11	11.43	0.04	56	1.12 (0.26)		
Frequency (days/week)	<3	11	433	0.26 [−0.09, 0.61]	0.23	31.48	0.0005	68	1.46 (0.14)		1.82 (0.18)
	≥3	4	163	−0.06 [−0.37, 0.25]	0.00	1.72	0.63	0	0.39 (0.70)		

IL-6, interleukin-6; CRP, C-reactive protein; TC, total cholesterol; TG, triglyceride; HDL, high density lipoprotein; LDL, low density lipoprotein; ASM, appendicular skeletal muscle mass; ASMI, appendicular skeletal muscle mass index; SM, skeletal muscle mass; BMI, body mass index; BF%, percentage of body fat; BIA, bioelectrical impedance analysis; DXA, dual energy X-ray absorptiometry; n, the number of participants; BMD, bone mineral density; SD, standard deviation.

*Inflammatory markers:* There were no significant relationships between inflammatory markers and age, intervention duration, intervention frequency, sarcopenia and obesity diagnostic indicators, and sarcopenia assessment methods.

*Lipid profiles*: There were no significant relationships between lipid profiles and age, intervention duration, intervention frequency, sarcopenia assessment methods, and sarcopenia and obesity diagnostic indicators.

*Muscle mass*: Muscle mass increased significantly in people <65 years (SMD = 0.52, 95% CI: 0.14 to 0.90, *p* = 0.007, *I* ^*2*^ = 0%). Muscle mass increased significantly when the obesity diagnostic indicator was BMI (SMD = 0.36, 95% CI: 0.11 to 0.62, *p* = 0.005, *I* ^*2*^ = 29%). Concerning the training protocol, a greater effect on muscle mass was observed when intervention duration was less than 12 weeks as compared with longer durations (SMD = 0.46, 95% CI: 0.23 to 0.69, *p* = 0.0001, *I* ^*2*^ = 0%).

## Discussion

In this systematic review and meta-analysis, we analyzed 12 studies (including 11 randomized controlled trials and one non-randomized controlled trial) to compare the effects of three exercise modes (AT, RT, and MT) on body composition, muscle mass, muscle strength, physical performance and hematological parameters in older people with SO. Our results found that AT could significantly decrease BW and BMI. RT could improve BF%, ASMI, ASM, HG, and KES, and, MT could improve BMI, BF%, ASMI, and GS in older people with SO. PA could significantly increase IGF-1, but all exercise modes (AT, RT, and MT) had no effects on other inflammatory markers and lipids profile markers in older people with SO.

An epidemiological study showed that the decrease of skeletal muscle mass in middle-aged and elderly women over 50 years old was greater than that in men. The decrease in estrogen level was the most important physiological characteristic of postmenopausal women. This change would lead to secondary body composition changes (Wang et al., 2016). The fat mass of the human body would increase significantly after the age of 45, especially in the elderly stage of women, due to the influence of physiological factors and the reduction of physical activity, fat mass was easier to accumulate, and abdominal fat increases obviously, resulting in obesity (Bahrami et al., 2006). Therefore, it was recommended that older people with SO, especially women, improve their body composition and health through gradual and regular PA.

BW, BF%, and BMI are obesity-related indicators associated with insulin resistance (Kurniawan et al., 2018). Progression of SO is often accompanied by changes in body composition. Obesity accompanies a chronic inflammatory state and plays a negative role in SO progression (Wijesinghe et al., 2021). Therefore, improving body composition is crucial for older people with SO. Also, studies indicated that PA is an effective intervention component to ameliorate the adverse effects caused by aging and obesity (Vincent et al., 2012). Consistent with our results, previous studies have shown that AT decreases BW while RT and MT improve BF% in older people with SO (Hsu et al., 2019). Wang et al. (2019) and Chen et al. (2017) also showed that MT (aerobic combined with resistance training for 8 weeks, once or twice a week) significantly improved BMI and BF% in older people with SO.

SM and ASM are measures of lean muscle mass measured by BIA or DXA. Our findings agreed with Hsu et al. (2019) and Hita-Contreras et al. (2018), showing that none of the exercise modes changed the SM values. In our study, other exercise modes had no effect on ASM except RT. Although AT, RT, and MT may not completely reverse the loss of SM and ASM, regular PA helps slow down many age-related mitochondrial markers of muscle, potentially delaying the process of muscle loss (Koltai et al., 2012).

ASMI personalizes measures of muscle mass in older people with SO, adjusting ASM by height or weight. Our results found that RT and MT significantly improved ASMI. RT improves muscle strength, mass, and neuromuscular function (Cadore et al., 2014). Chelly et al. (2009) observed that the first 8 weeks of RT usually improves neural adaptation rather than changing muscle structure in novice weight lifters. Thus, we speculate that different training protocols might effect muscle mass differently. We found improved muscle mass of SO people when intervention duration lasted less than 12 weeks in a subsample of people aged <65 with obesity status determined by BMI. This finding persisted regardless of the sarcopenia assessment method, BMI levels, and intervention frequency. This finding was inconsistent with the study by Chen et al. (2021) found that RT 1–2 times per week for ≥12 weeks could improve muscle mass . Contrary to Chen et al. (2021) study, we speculated that duplicate inclusion of the subgroup analysis less than 12 weeks would affect the final results of the meta-analysis. We extracted subgroup data for different modes of PA and different indicators. For example, two studies with fewer than 12 weeks of exercise duration contained nine subgroup combinations (Chen et al., 2017; Wang et al., 2019). Six studies with more than 12 weeks duration (Kim et al., 2016; Park et al., 2017; Chiu et al., 2018; Liao et al., 2018; Li et al., 2020; Lee et al., 2021) contained six subgroup combinations. Eleven studies had an exercise frequency of fewer than three times per week, and four studies had more than three times per week. A significant imbalance in the number of studies analyzed by subgroups may affect the final results. Therefore, we suggest future studies of PA in older people with SO last longer than 12 weeks and with an exercise frequency of three or more times per week.

Muscle strength declines with age at an average rate of 2%–4% per year, 2–5 times faster than muscle mass loss (Mitchell et al., 2012). Muscle strength and physical performance are predictors of disability and hospitalization in older people (Legrand et al., 2014). Lu et al. (2020) showed that HG strength correlates with arm and leg strength and represents whole-body muscle strength. KES is a method for assessing lower limb strength and predicting falling risk (Bobowik and Wiszomirska, 2020). Consistent with Hsu et al. (2019), our study showed that RT significantly improved both HG and KES, and MT improved GS. MT combines the dual advantages of AT and RT and should have a better effect on muscle strength than RT in improving muscle strength. However, our results did not find that MT significantly improved the subjects’ muscle strength, which was inconsistent with previous studies. A meta-analysis by Lu et al. (2021) indicated that RT and MT had the same effect on muscle strength in people with sarcopenia. This increase in muscle strength may be due to the complex training in Lu et al.(2021) study was aerobic combined resistance exercise and balance and gait training. However, no studies have explored the comparison of RT and MT on muscle strength in older people with SO. Thus, researchers should conduct more research to compare these two modes of exercise in people with SO. Consistent with Lu et al.(2021) results, MT could improve GS, but inconsistent with our study, they also pointed out that RT could improve GS. This difference may be that the longest training time for RT on GS in Lu et al.(2021) study was 24 weeks, while in our study, the longest RT time for GS was 12 weeks. Increased neural activity in areas of the brain associated with cognition and memory was one of the potential mechanisms for action (Hansen, 2014). Therefore, we hypothesized that with longer training durations, the proficiency of the movement increases, and the training difficulty of MT becomes more complex than that of AT and RT. This effect causes the whole body to promote increases in GS.

IGF-1 positively effects the body as it mediates growth hormone and anabolic responses in many cells and tissues. Conversely, chronic inflammation and hyperlipidemia have adverse effects on various tissues and cellular functions, including CVD progression. Our study indicated that PA significantly improved IGF-1, and none of the exercise modes changed inflammatory markers and lipid profiles. As shown by Li et al. (2021), exercise induced the secretion of large amounts of IGF-1 from liver and skeletal muscle and triggered a series of downstream responses, such as activation of skeletal muscle satellite cells to promote myogenic cell proliferation and differentiation, and inhibition of cell expression of collagen to reduce skeletal muscle fibrosis. In conclusion, skeletal muscle cells could produce IGF-1 in response to exercise stimuli, which played a protective role in maintaining muscle mass and function. Wang et al. (2019) demonstrated that in older people with SO, 8-weeks of RT could reduce IL-6, and 8-weeks of RT and MT increased IGF-1. Park et al. (2017) found that 24 weeks of MT significantly improved TC and LDL in older people with SO. By contrast, Hsu et al. (2019) showed that PA, regardless of exercise mode, did not affect CRP, TC, TG, and HDL in older people with SO. Therefore, the effect of PA on inflammatory markers and lipids profiles in people with SO is equivocal. We speculate that differences in subject characteristics and training protocols are responsible for these findings. In subgroup analyses, we expected to see the positive effects of different moderators on inflammatory markers and lipid profiles.

Contrary to expectations, we did not find a significant relationship between moderators, inflammatory markers, and lipid profiles. We infer that this may be related to the limited number of studies included in the meta-analyses reviewed. Thus, more research should explore different exercise modes on inflammatory markers and lipid profiles in older people with SO.

In the subgroup analysis, some of the results showed high heterogeneity. The reasons for this may be composed of the following points. First, the diagnostic criteria for SO varied by region, race, age, and measurement tools, and the diagnostic criteria for SO were a combination of diagnostic criteria for sarcopenia and diagnostic criteria for obesity, but the prevalence of the SO varied widely using different combinations. Second, the inconsistent baseline characteristics of the older people with SO in the included studies may have led to differences in the improvement effect of different exercise modes on various indicators. Finally, the diversity of exercise intervention protocols and the inconsistency of quality monitoring during the intervention resulted in high heterogeneity.

### Strengths and limitations

This systematic review and meta-analysis comprehensively assessed the effects of different modes of PA (AT, RT, and MT) in older people with SO. The strength of this study is the rigorous screening where we excluded studies of subjects without SO, studies where SO subjects were treated with nutritional supplements, and studies where SO subjects were diagnosed with chronic diseases. Our findings were comprehensive and consisted of the changes in body composition, muscle mass, muscle strength, physical performance, and hematological parameters, including the effect of PA on IGF-1 in older people with SO.

This study also had some limitations. We included studies that recruited older people with osteosarcopenic obesity. However, researchers know little about how osteoporosis may affect PA in older people with SO. In our subgroup analysis, after PA intervention, people without osteoporosis significantly decreased BF% compared with older people with osteoporosis. Postmenopausal women lost the protective effect of estrogen, osteoclasts, and bone absorption were enhanced, resulting in sparse trabecular bone and reduced bone mass, which increased the difficulty of PA intervention when co-occurring with SO (Li et al., 2020). Therefore, osteoporosis can affect the effect of exercise on SO. Limited to the number of included studies, we only explored the influence of the efficacy of exercise on BF%. More RCTs are needed to study this relationship in the future. The intervention duration for studies in this systematic review and meta-analysis was shorter than 24 weeks, which may limit changes in muscle mass and hematological parameters. More studies are needed to investigate the effects of long-term PA on SO. Based on strict inclusion and exclusion criteria, the number of eligible studies was small, and the subjects were predominantly female, limiting the general applicability of the meta-analysis results.

## Conclusion

Our results illustrated the importance of PA in the management of SO in older people. Different modes of exercise selectively improve body composition (BW, BMI, and BF%), muscle mass (ASMI and ASM), muscle strength (HG and KES), physical performance (GS), and hematological parameters (IGF-1) in older people with SO. In particular, AT decreases BW and BMI; RT improves BF%, ASMI, ASM, HG, and KES; and MT improves BMI, BF%, ASMI, and GS. PA increased IGF-1. These findings require additional high-quality RCTs with longer intervention duration to confirm these benefits of PA in older people with SO.

## Data Availability

The original contributions presented in the study are included in the article/Supplementary Material, further inquiries can be directed to the corresponding authors.
