# Supplementary material for: Effects of three modes of physical activity on physical fitness and hematological parameters in older people with sarcopenic obesity: A systematic review and meta-analysis
Source: Front Physiol. 2022 Aug 25;13:917525. doi: 10.3389/fphys.2022.917525 (PMC9458075; doi:10.3389/fphys.2022.917525)
Supplement: Supplementary file 1 [file Table1.DOC]

**Table 1: Characteristics of Included Studies**

| **Study** | **Sample size** (ETG/CG) | **Gender**  (n: male /female) | **Age** (ETG/CG) | **Sarcopenia diagnostics**  (Indicator, Cut-points, Source) | **Obesity diagnostics**  (Indicator, Cut-points, Source) | **Intervention** | | | | **Control group** | **Outcome** |
| --- | --- | --- | --- | --- | --- | --- | --- | --- | --- | --- | --- |
|  |  |  |  |  |  | **Mode** | **Training movement** | **Intensity** | **Duration** days/week(weeks) |  |  |
| (Huang et al., 2017) | 18/17 | 0/35 | 68.89±4.91/  69.53±5.09 | SMM/weight²*100% by BIA<27.6  (Janssen) | BF% by BIA>30%  (Liu) | RT: Elastic band | RT: Muscle group training included shoulders, arms, lower limbs, chest, and abdomen | 3 sets/10 reps | 3(12) | Education | BW, BMI, BF%, TG, HDL, LDL, TC, CRP |
| (Vasconcelos et al., 2016) | 14/14 | 0/28 | 72±4.6/  72±3.6 | HG≤21 kg  (Fried) | BMI≥30kg/m² (Vasconcelos) | RT: Elastic band | RT: Knee exercises, hip exercises, and mini-squats | 2-3 sets/12 reps (40-60% 1RM) for knee exercises; 2-3 sets/12 reps (1-3kg) for hip exercises; 2-3 sets/10 reps (1-3kg) for mini-squats | 2(10) | Non-  exercise | KES, GS |
| (Liao et al., 2018) | 33/23 | 0/56 | 66.67±4.54/  68.32±6.05 | SMM/weight*100% by BIA<27.6% (Janssen) | BF% by BIA>30%  (Liu) | RT: Elastic band | RT: Upper body exercises included seated chest press, seated row, seated shoulder press; Lower body exercises included knee extension, knee flexion, hip flexion, and hip extension | 3 sets/10 reps; RPE=13 | 3(12) | Non-  exercise | BF%, SM, HG, KES, GS |
| (Liao et al., 2017) | 25/21 | 0/46 | 66.39±4.49  /68.42±5.86 | SMM/height² by BIA<7.15kg/m² (EWGSOP-2010) | BF% by BIA>30%  (Baumgartner) | RT: Elastic band | RT: Seated chest press, seated row, seated shoulder press, concentric–eccentric hip circumduction, leg press, leg curl | 3 sets/1-20 reps;  RPE=13 | 3(12) | Non-  exercise | BF%, HG, KES, GS |
| (Chiu et al., 2018) | 36/34 | 35/35 | 79.64±7.36/  80.15±8.26 | SMM/weight*100% by BIA, M: ≤37.15%,  F: ≤32.26%  （Janssen） | BF%, F: ≥29%, M: ≥40%  （Ko） | RT: Sandbag and grip ball | RT: Upper extremities training that targeted the biceps, deltoids, grip, and pinch; Lower extremities training included leg extension, leg flexion, calf raises, stepping forward and sideward | 3 sets/4–10 reps | 2(12) | Non-  exercise | BF%, ASM, HG |
| (Park et al., 2017) | 25/25 | 0/50 | 73.5±7.1/  74.7±5.1 | ASM/weight*100% by BIA<25.1%  (Lim) | BMI≥25.0kg/m²  (Lim) | RT: Elastic band  AT:  Walking | RT: elbow flexion, wrist flexion, shoulder flexion, lateral raise, front raise, chest press, reverse flies, side band, dead lift, squat, leg press, ankle plantar flexion AT: sideways, backward, forward walking, slow and fast indoor walking | All 50-80 min RT:2-3sets/8-15reps,20-30 min/session; AT:30-50min/session with the RPE=13-17 | RT: 3(24) AT: 5(24) | Education | BF%, ASM, HG, GS, TG, HDL, LDL, TC, CRP |
| (Chen et al., 2017) | RT:15 AT:15 RT+AT:15 CG:15 | 10/50 | RT:68.9±4.4 AT:69.3±3.0 RT+AT:  68.5±2.7 CG:68.6±3.1 | ASM/Weight*100%, M: ≤32.5%, F: ≤25.7%  （Chung） | BMI≥ 25 kg/m²  (WHO);  VFA≥ 100cm²  (Lu） | RT: Weight-  training equipment  AT: dance steps class | RT: shoulder presses, bicep curls, triceps curls, bench presses, deadlifts, leg swings, squats, standing rows, unilateral rows, and split front squats. AT: stepping on the spot, knee lifts, high knee running, rowing arm swings, arm swings, twist steps, arm raises, squats, V steps, mambo steps, diamond steps, and point step jumps | RT: 3 sets/8–12 reps  AT: moderate intensity (> 3 metabolic equivalents) | RT: 2(8) AT: 2(8) RT+AT: 1(8) | Non-  exercise | BW, BMI, BF%, ASMI, SM, HG, KES, IGF-1 |
| (Kim et al., 2016) | RT+AT:35  CG:34 | 0/69 | All:  81.4±4.3/  81.1±5.1 | SMM/height² by DXA<5.67 kg/m² or HG< 17.0 kg or GS< 1.0 m/s（Kim-2016） | BF% by DXA≥32%  (Kim-2016) | RT: Weight machines, Elastic band AT: Stationary bicycle | RT: toe raises, heel raises, knee lifts,and knee extension, hip flexion, seated row, leg press, abduction, leg extension, and abdominal crunch  AT: Stationary bicycle | RT: 1-3/10 reps AT: 12 minutes | RT:2(12) AT:2(12) | Education | BW, BF%, ASM, HG, KES, GS, TG, TC, IL-6, CRP |
| (Wang et al., 2019) | RT:20 AT:20 RT+AT:20 CG:20 | 43/37 | RT:65.1±3.4 AT:64.2±3.0 RT+AT:63.6±5.2  CG:64.1±2.8 | ASM by DXA, M: <7 kg/m², F:<5.4 kg/m²; HG, M: <26kg, F:<18kg; (FNIH) | BMI, M: < 0.789, F: < 0.512  (FNIH) | / | RT: Hands, feet, abdomen, pelvis and back muscle training  AT: Stepping, knee lift, leg lift, arm swing, arm lift, diamond step and dot step jump | RT:3-5sets/10-15reps AT: 40%-60%  VO_2_max  RT+AT: RT for 10 min, AT for 20 min | RT: 2(8) AT: 2(8) RT+AT: 2(8) | Non-  exercise | BW, BMI, BF%, ASMI, HG, KES, IL-6, IGF-1 |
| (Li et al., 2020) | RT+AT:15 CG:15 | / | RT+AT:  63.87±3.56 CG:64.93±  3.84 | ASM/height² by DXA, M: ≤7.0kg/m²,  F: ≤5.4kg/m²  (AGWS-2013) | BF% by DXA, M: ≥25%, F: ≥35% (WHO) | RT: Elastic band  AT: Speed walking | RT: Major muscle groups training of limbs and trunk AT: Speed walking | RT:1-3sets/10reps, RPE=5-6/10 AT: 60%-80% HRmax,  RPE =5-6/10 | RT: 3(12) AT: 5(12) | Non-  exercise | BF%, ASM |
| (Banitalebi et al., 2021) | RT:32 CG:31 | 0/63 | RT:64.11±  3.81 CG:64.05±  3.35 | SMI by DXA ≤ 28% or ≤ 7.76 kg/m²; GS ≤1m/s (Newman) | BF% by DXA≥32%; BMI by DXA >30kg/m²  (ASBP) | RT: Elastic band | RT: major muscle groups training (legs, back, abdomen, chest, shoulders, and arms) | RT: 1-2/12 reps | RT: 3(12) | Non-  exercise | BW, BMI, BF% |
| (Lee et al., 2021) | RT:15 CG:12 | 0/27 | RT:70.13±4.41 CG:71.82±5.23 | ASM/height² by DXA < 5.67 kg/m² and HG < 20 kg or GS < 0.8 m/s  (EWGSOP-2010) | BF% by DXA,>35%  (Li) | RT: Elastic band | RT: major muscle groups training (shoulders, arms, lower limbs, chest, and abdomen) | RT: 3 set/10 reps | RT: 3(12) | Non-  exercise | BF%, SM, HG, GS |

ETG: exercise training group; CG: control group; RT: resistance training; AT: aerobic training; reps: repetition; RPE: rated perceived exercise; 1RM: one repetition maximum; W: week; BIA: bioelectrical impedance analysis; DXA: dual energy X-ray absorptiometry; BW: body weight**;** BMI: body mass index; BF%: percentage body fat; SM: skeletal muscle mass; ASM: appendicular skeletal muscle mass; ASMI: appendicular skeletal muscle mass index; HG: handgrip strength; GS: gait speed; KES: knee extension strength; TG: triglyceride; TC: total cholesterol; HDL: high density lipoprotein; LDL: low density lipoprotein; IL-6: interleukin-6; CRP: C-reactive protein; IGF-1: insulin-like growth factor 1; HRmax: maximal heart rate; EWGSOP: European Working Group on Sarcopenia in Older People; FNIH: Foundation for the National Institutes of Health; AWGS: Asian Working Group for Sarcopenia; ASBP: the American Society of Bariatric Physicians; Age is expressed as mean ± standard deviation;

| **Table 2a** Different indicators and cut-off points in defining sarcopenia | | | | |
| --- | --- | --- | --- | --- |
| **Diagnosis Criteria** | **Target District** | **Cut-off Points** | | |
|  |  | **Muscle mass** | **Muscle strength** | **Muscle performance** |
| EWGSOP-2010  (Cruz-Jentoft et al., 2010) | countries from Europe | ASM/height² by DXA:(M:<7.26 kg/m², F:<5.50kg/m²)； or SM/height² by BIA:(M:≤8.87kg/m², F:≤6.42kg/m²) | HG: (M:<30 kg, F:<20 kg) | GS (4m): < 0.8m/s;  or GS (6m): < 1m/s  or SPPB: ≤ 8 |
| AWGS-2013  (Chen et al., 2014) | countries from Asia | ASM/height² by DXA: (M:≤7.0 kg/m², F:≤5.4kg/m²)； or ASM//height² by BIA:(M: ≤7.0 kg/m², F: ≤5.7kg/m²) | HG: (M:<26 kg, F:<18 kg) | GS (6m): <0.8 m/s |
| FNIH  (Studenski et al., 2014) | United States | ASM/BMI by DXA: (M<0.789, F<0.512) | HG: (M<26kg, F<16kg) | / |
| Janssen  (Janssen et al., 2002) | United States | [(height^2^/BIA-resistance*0.401) +3.825(gender)+0.071(age)+ 5.102]/body mass*100] <1 standard deviations of a young reference population | / | / |
| Chung  (Chung et al., 2013) | Korea | ASM/weight*100% by DXA, M: ≤32.5%, F: ≤25.7% | / | / |
| Newman  (Newman et al., 2003) | United States | F:ALM (kg)=-13.19+14.75*height (m)+0.23*total fat mass(kg) M:ALM (kg)=-22.48+24.14*height(m)+0.21*total fat mass(kg), the 20th percentile of the distribution of residuals | / | / |
| Lim  (Lim et al., 2010) | Korea | ASM/height² by DXA:(M<7.09kg/m²,F<5.27kg/m²)； or ASM/weight*100% by DXA:(M<29.9%, F<25.1%) | / | / |
| Kim  (Kim et al., 2016) | Japan | SM/height² by DXA < 5.67 kg/m² | HG: <17.0 kg | GS (5m): <1.0 m/s |
| Fried  (Fried et al., 2001) | United States | weight loss>10 pounds  or ≥5% of body weight of the previous year | HG: lowest 20% (by gender, BMI) | GS: slowest 20% (by gender, height) |

BIA: bioelectrical impedance analysis; DXA: dual energy X-ray absorptiometry; BMI: body mass index; SM: skeletal muscle mass (kg); ASM: appendicular skeletal muscle mass; ALM: appendicular lean mass; HG: handgrip strength; GS: gait speed; SPPB: the short physical performance battery; TUG: time up and go test; EWGSOP: European Working Group on Sarcopenia in Older People; FNIH: Foundation for the National Institutes of Health; AWGS: Asian Working Group for Sarcopenia; M: male; F: female

**Table 2b Different indicators and cut-off points in defining obesity**

| **Diagnosis Criteria** | **Target District** | **Cut-off Points** |
| --- | --- | --- |
| Deurenberg  (Deurenberg et al., 1998) | America, Caucasia, China, Ethiopia, Indonesia, Polynesia and Thailand. | BF% by BIA>30% |
| Ko  (Ko et al., 2001) | China | BF% by BIA,F: ≥29%，M: ≥40% |
| Vasconcelos  (Vasconcelos et al., 2016) | Brazil | BMI≥30kg/m² |
| Baumgartner  (Baumgartner, 2000) | New Mexico | BF% by BIA>30% |
| Lim  (Lim et al., 2010) | Korea | VFA by abdominal CT>100cm² |
| FNIH  (Studenski et al., 2014) | / | BMI, M:<0.789, F:< 0.512 |
| Kim  (Kim et al., 2016) | Japan | BF% by DXA, ≥ 32% |
| WHO  (Use and Anthropometry, 1995) | Asia | BF% by DXA, M: ≥ 25%, F: ≥ 35%; BMI ≥ 25 kg/m² |
| Li  (Li et al., 2012) | China | BF% by BIA, M: ≥ 25%, F: ≥ 35% |
| ASBP  (Ilich et al., 2016) | United States | BF% by DXA ≥ 32% |

BIA: bioelectrical impedance analysis; DXA: dual energy X-ray absorptiometry; BF%: body fat percentage; VFA: visceral fat area; CT: computed tomography; BMI: body mass index; M: male; F: female; FNIH: Foundation for the National Institutes of Health; WHO: World Health Organization; ASBP: American Society of Bariatric Physicians

**Table 3 PEDro Criteria and Scores of Included Studies**

| **study** | **Eligibility criteria** | **Random allocation** | **Concealed allocation** | **Baseline similar** | **Blinding (subject)** | **Blinding (therapists)** | **Blinding (Assessor)** | **Measure for>85%** | **Intention-to-Treat Analysis** | **Group comparison** | **Point measures** | **Total score (0-10)** |
| --- | --- | --- | --- | --- | --- | --- | --- | --- | --- | --- | --- | --- |
|
| (Huang et al., 2017) | yes | 1 | 1 | 1 | 0 | 0 | 1 | 1 | 1 | 1 | 1 | 8 |
| (Vasconcelos et al., 2016) | yes | 1 | 1 | 1 | 0 | 0 | 1 | 1 | 1 | 1 | 1 | 8 |
| (Liao et al., 2018) | yes | 1 | 1 | 1 | 0 | 0 | 1 | 1 | 1 | 1 | 1 | 8 |
| (Liao et al., 2017) | yes | 1 | 1 | 1 | 1 | 1 | 1 | 1 | 1 | 1 | 1 | 10 |
| (Chiu et al., 2018) | yes | 0 | 0 | 1 | 1 | 1 | 0 | 0 | 0 | 1 | 1 | 5 |
| (Park et al., 2017) | yes | 1 | 0 | 1 | 0 | 0 | 1 | 1 | 1 | 1 | 1 | 7 |
| (Chen et al., 2017) | yes | 1 | 0 | 1 | 0 | 0 | 1 | 0 | 0 | 1 | 1 | 5 |
| (Kim et al., 2016) | yes | 1 | 1 | 1 | 0 | 0 | 0 | 1 | 0 | 1 | 1 | 6 |
| (Wang et al., 2019) | yes | 1 | 1 | 1 | 0 | 0 | 0 | 1 | 0 | 1 | 1 | 6 |
| (Li et al., 2020) | yes | 1 | 1 | 1 | 0 | 0 | 0 | 1 | 1 | 1 | 1 | 7 |
| (Banitalebi et al., 2021) | yes | 1 | 1 | 1 | 0 | 0 | 0 | 1 | 1 | 1 | 1 | 7 |
| (Lee et al., 2021) | yes | 1 | 1 | 1 | 0 | 0 | 1 | 1 | 1 | 1 | 1 | 8 |

**PEDro: Physiotherapy Evidence Database; 1: meet the standard; 0: not meet the standard**

**Table 4 Influence of moderator variables in the effect of physical activity on Inflammatory markers, Lipid profiles, BMD and muscle mass**

| **Variable** | **Subgroup** | **Studies** | **n** | **Effect Size with 95% Confidence Interval** | **Heterogeneity** | | | **Test overall effects. Z(p)** | **Test for Subgroup Difference.Chi²(p)** |
| --- | --- | --- | --- | --- | --- | --- | --- | --- | --- |
|  |  |  |  |  | **Chi²** | **P** | **I²** |  |  |
| **Inflammatory markers (IL-6 and CRP)** | | | | | | | | | |
| Age (years) | < 70 | 4 | 155 | -0.06 [-0.37, 0.26] | 0.33 | 0.95 | 0 | 0.36(0.72) | 0.86(0.35) |
|  | ≥ 70 | 3 | 188 | 0.14 [-0.14, 0.43] | 0.71 | 0.70 | 0 | 0.98(0.33) |  |
| Intervention duration (weeks) | < 12 | 3 | 120 | -0.06 [-0.42, 0.29] | 0.33 | 0.85 | 0 | 0.35(0.73) | 0.62(0.43) |
|  | ≥ 12 | 4 | 223 | 0.11 [-0.15, 0.38] | 0.95 | 0.81 | 0 | 0.86(0.39) |  |
| Frequency (days/week) | < 3 | 5 | 258 | 0.07 [-0.17, 0.32] | 1.77 | 0.78 | 0 | 0.60(0.55) | 0.13(0.72) |
|  | ≥ 3 | 2 | 85 | -0.02 [-0.44, 0.41] | 0.01 | 0.93 | 0 | 0.07(0.94) |  |
| Sarcopenia Assessment method | BIA | 2 | 85 | -0.02 [-0.44, 0.41] | 0.01 | 0.93 | 0 | 0.07(0.94) | 0.13(0.72) |
|  | DXA | 5 | 258 | 0.07 [-0.17, 0.32] | 1.77 | 0.78 | 0 | 0.60(0.55) |  |
| Sarcopenia diagnostic indicator | SM | 3 | 173 | 0.15 [-0.15, 0.45] | 0.74 | 0.69 | 0 | 0.97(0.33) | 0.80(0.37) |
|  | ASM | 4 | 170 | -0.05 [-0.35, 0.26] | 0.37 | 0.95 | 0 | 0.29(0.77) |  |
| Obesity diagnostic indicator | BF% | 3 | 173 | 0.15 [-0.15, 0.45] | 0.74 | 0.69 | 0 | 0.97(0.33) | 0.80(0.37) |
|  | BMI | 4 | 170 | -0.05 [-0.35, 0.26] | 0.37 | 0.95 | 0 | 0.29(0.77) |  |
| **Lipid profiles (TC, TG, HDL LDL)** | | | | | | | | | |
| Age (years) | < 70 | 4 | 140 | 0.13 [-0.20, 0.46] | 1.11 | 0.77 | 0 | 0.77(0.44) | 1.16(0.28) |
|  | ≥ 70 | 6 | 338 | -0.09 [-0.30, 0.13] | 3.54 | 0.62 | 0 | 0.79(0.43) |  |
| Sarcopenia assessment method | BIA | 8 | 340 | -0.02 [-0.24, 0.19] | 4.96 | 0.66 | 0 | 0.23(0.82) | 0.00(0.97) |
|  | DXA | 2 | 138 | -0.02 [-0.35, 0.32] | 0.85 | 0.36 | 0 | 0.11(0.91) |  |
| Sarcopenia diagnostic indicator | SM | 6 | 278 | 0.06 [-0.18, 0.29] | 2.34 | 0.80 | 0 | 0.47(0.64) | 1.04(0.31) |
|  | ASM | 4 | 200 | -0.13 [-0.41, 0.14] | 2.42 | 0.49 | 0 | 0.94(0.35) |  |
| Obesity diagnostic indicator | BF% | 5 | 243 | 0.08 [-0.17, 0.34] | 2.01 | 0.73 | 0 | 0.64(0.52) | 2.51(0.11) |
|  | BMI | 3 | 150 | -0.25 [-0.57, 0.07] | 0.50 | 0.78 | 0 | 1.51(0.13) |  |
| Intervention duration (weeks) | ≤ 12 | 6 | 278 | 0.06 [-0.18, 0.29] | 2.34 | 0.80 | 0 | 0.47(0.64) | 1.04(0.31) |
|  | > 12 | 4 | 200 | -0.13 [-0.41, 0.14] | 2.42 | 0.49 | 0 | 0.94(0.35) |  |
| Frequency (days/week) | ≤ 3 | 6 | 278 | 0.06 [-0.18, 0.29] | 2.34 | 0.80 | 0 | 0.47(0.64) | 1.04(0.31) |
|  | > 3 | 4 | 200 | -0.13 [-0.41, 0.14] | 2.42 | 0.49 | 0 | 0.94(0.35) |  |
| **BMD** | | | | | | | | | |
| T-score | > -1 SD | 12 | 530 | -0.52 [-0.70, -0.35] | 13.81 | 0.24 | 20 | 5.84(< 0.00001) | 0.51(0.48) |
|  | ≤ -1 SD | 3 | 119 | -0.37 [-0.74, -0.01] | 3.35 | 0.19 | 40 | 2.00(0.05) |  |

| **Variable** | **Subgroup** | **Studies** | **n** | **Effect Size with 95% Confidence Interval** | **Heterogeneity** | | | | **Test overall effects.**  **Z(p)** | **Test for Subgroup Difference.Chi²(p)** |
| --- | --- | --- | --- | --- | --- | --- | --- | --- | --- | --- |
|  |  |  |  |  | **Tau²** | **Chi²** | **P** | **I²** |  |  |
| **SM, ASM and ASMI** | | | | | | | | | | |
| Age (years) | < 65 | 3 | 110 | 0.52 [0.14, 0.90] | 0.00 | 1.04 | 0.59 | 0 | 2.68(0.007) | 3.15(0.08) |
|  | ≥ 65 | 12 | 486 | 0.08 [-0.22, 0.38] | 0.17 | 28.96 | 0.002 | 62 | 0.54(0.59) |  |
| Sarcopenia Assessment method | BIA | 13 | 539 | 0.20 [-0.09, 0.49] | 0.18 | 33.50 | 0.0008 | 64 | 1.33(0.18) | 0.62(0.43) |
|  | DXA | 2 | 57 | -0.05 [-0.61, 0.50] | 0.02 | 1.13 | 0.29 | 11 | 0.19(0.85) |  |
| Obesity diagnostic indicator | BF% | 5 | 246 | -0.20 [-0.64, 0.24] | 0.16 | 11.42 | 0.02 | 65 | 0.88(0.38) | 4.67(0.03) |
|  | BMI | 10 | 350 | 0.36 [0.11, 0.62] | 0.05 | 12.64 | 0.18 | 29 | 2.80(0.005) |  |
| BMI levels | < 27 kg/m² | 10 | 390 | 0.09 [-0.26, 0.44] | 0.21 | 26.36 | 0.002 | 66 | 0.51(0.61) | 0.64(0.42) |
|  | ≥ 27 kg/m² | 5 | 206 | 0.30 [-0.08, 0.68] | 0.08 | 7.19 | 0.13 | 44 | 1.57(0.12) |  |
| Intervention duration (weeks) | < 12 | 9 | 300 | 0.46 [0.23, 0.69] | 0.00 | 7.72 | 0.46 | 0 | 3.87(0.0001) | 9.28(0.002) |
|  | ≥ 12 | 6 | 296 | -0.20 [-0.56, 0.15] | 0.11 | 11.43 | 0.04 | 56 | 1.12(0.26) |  |
| Frequency (days/week) | < 3 | 11 | 433 | 0.26 [-0.09, 0.61] | 0.23 | 31.48 | 0.0005 | 68 | 1.46(0.14) | 1.82(0.18) |
|  | ≥ 3 | 4 | 163 | -0.06 [-0.37, 0.25] | 0.00 | 1.72 | 0.63 | 0 | 0.39(0.70) |  |

IL-6: interleukin-6; CRP: C-reactive protein; TC: total cholesterol; TG: triglyceride; HDL: high density lipoprotein; LDL: low density lipoprotein; ASM: appendicular skeletal muscle mass; ASMI: appendicular skeletal muscle mass index; SM: skeletal muscle mass; BMI: body mass index; BF%: percentage of body fat; BIA: bioelectrical impedance analysis; DXA: dual energy X-ray absorptiometry; n: the number of participants; BMD: bone mineral density; SD: standard deviation

**References**

Banitalebi, E., Ghahfarrokhi, M.M., and Dehghan, M. (2021). Effect of 12-weeks elastic band resistance training on MyomiRs and osteoporosis markers in elderly women with Osteosarcopenic obesity: a randomized controlled trial. *BMC Geriatr* 21(1)**,** 433. doi: 10.1186/s12877-021-02374-9.

Baumgartner, R. (2000). Body composition in healthy aging. *Annals of the New York Academy of Sciences* 904**,** 437-448. doi: 10.1111/j.1749-6632.2000.tb06498.x.

Chen, H.T., Chung, Y.C., Chen, Y.J., Ho, S.Y., and Wu, H.J. (2017). Effects of Different Types of Exercise on Body Composition, Muscle Strength, and IGF-1 in the Elderly with Sarcopenic Obesity. *J Am Geriatr Soc* 65(4)**,** 827-832. doi: 10.1111/jgs.14722.

Chen, L.K., Liu, L.K., Woo, J., Assantachai, P., Auyeung, T.W., Bahyah, K.S., et al. (2014). Sarcopenia in Asia: Consensus Report of the Asian Working Group for Sarcopenia. *Journal of the American Medical Directors Association* 15(2)**,** 95-101. doi: 10.1016/j.jamda.2013.11.025.

Chiu, S.C., Yang, R.S., Yang, R.J., and Chang, S.F. (2018). Effects of resistance training on body composition and functional capacity among sarcopenic obese residents in long-term care facilities: a preliminary study. *BMC Geriatr* 18(1)**,** 21. doi: 10.1186/s12877-018-0714-6.

Chung, J., Kang, H., Lee, D., Lee, H., and Lee, Y. (2013). Body composition and its association with cardiometabolic risk factors in the elderly: a focus on sarcopenic obesity. *Archives of gerontology and geriatrics* 56(1)**,** 270-278. doi: 10.1016/j.archger.2012.09.007.

Cruz-Jentoft, A., Baeyens, J., Bauer, J., Boirie, Y., Cederholm, T., Landi, F., et al. (2010). Sarcopenia: European consensus on definition and diagnosis: Report of the European Working Group on Sarcopenia in Older People. *Age and ageing* 39(4)**,** 412-423. doi: 10.1093/ageing/afq034.

Deurenberg, P., Yap, M., and van Staveren, W. (1998). Body mass index and percent body fat: a meta analysis among different ethnic groups. *International journal of obesity and related metabolic disorders : journal of the International Association for the Study of Obesity* 22(12)**,** 1164-1171. doi: 10.1038/sj.ijo.0800741.

Fried, L., Tangen, C., Walston, J., Newman, A., Hirsch, C., Gottdiener, J., et al. (2001). Frailty in older adults: evidence for a phenotype. *The journals of gerontology. Series A, Biological sciences and medical sciences* 56(3)**,** M146-156. doi: 10.1093/gerona/56.3.m146.

Huang, S.W., Ku, J.W., Lin, L.F., Liao, C.D., Chou, L.C., and Liou, T.H. (2017). Body composition influenced by progressive elastic band resistance exercise of sarcopenic obesity elderly women: a pilot randomized controlled trial. *Eur J Phys Rehabil Med* 53(4)**,** 556-563. doi: 10.23736/s1973-9087.17.04443-4.

Ilich, J., Kelly, O., and Inglis, J. (2016). Osteosarcopenic Obesity Syndrome: What Is It and How Can It Be Identified and Diagnosed? *Current gerontology and geriatrics research* 2016**,** 7325973. doi: 10.1155/2016/7325973.

Janssen, I., Heymsfield, S., and Ross, R. (2002). Low relative skeletal muscle mass (sarcopenia) in older persons is associated with functional impairment and physical disability. *Journal of the American Geriatrics Society* 50(5)**,** 889-896. doi: 10.1046/j.1532-5415.2002.50216.x.

Kim, H., Kim, M., Kojima, N., Fujino, K., Hosoi, E., Kobayashi, H., et al. (2016). Exercise and Nutritional Supplementation on Community-Dwelling Elderly Japanese Women With Sarcopenic Obesity: A Randomized Controlled Trial. *J Am Med Dir Assoc* 17(11)**,** 1011-1019. doi: 10.1016/j.jamda.2016.06.016.

Ko, G., Tang, J., Chan, J., Sung, R., Wu, M., Wai, H., et al. (2001). Lower BMI cut-off value to define obesity in Hong Kong Chinese: an analysis based on body fat assessment by bioelectrical impedance. *The British journal of nutrition* 85(2)**,** 239-242. doi: 10.1079/bjn2000251.

Lee, Y.H., Lee, P.H., Lin, L.F., Liao, C.D., Liou, T.H., and Huang, S.W. (2021). Effects of progressive elastic band resistance exercise for aged osteosarcopenic adiposity women. *Experimental Gerontology* 147**,** 111272. doi: 10.1016/j.exger.2021.111272.

Li, L., Wang, C., Bao, Y., Peng, L., Gu, H., and Jia, W. (2012). Optimal body fat percentage cut-offs for obesity in Chinese adults. *Clinical and experimental pharmacology & physiology* 39(4)**,** 393-398. doi: 10.1111/j.1440-1681.2012.05684.x.

Li, S., Huang, L.P., Wang, L., Chen, Y.W., Li, L.K., Zhang, L., et al. (2020). Effects of 12 weeks aerobic exercise combined with high speed strength training on old adults with osteosarcopenic obesity syndrome. *Chinese Journal of Rehabilitation Medicine* 35(4)**,** 420-426. doi: 10.3969/j.issn.1001-1242.2020.04.007.

Liao, C.D., Tsauo, J.Y., Huang, S.W., Ku, J.W., Hsiao, D.J., and Liou, T.H. (2018). Effects of elastic band exercise on lean mass and physical capacity in older women with sarcopenic obesity: A randomized controlled trial. *Sci Rep* 8(1)**,** 2317. doi: 10.1038/s41598-018-20677-7.

Liao, C.D., Tsauo, J.Y., Lin, L.F., Huang, S.W., Ku, J.W., Chou, L.C., et al. (2017). Effects of elastic resistance exercise on body composition and physical capacity in older women with sarcopenic obesity. *Medicine (United States)* 96(23)**,** e7115. doi: 10.1097/MD.0000000000007115.

Lim, S., Kim, J., Yoon, J., Kang, S., Choi, S., Park, Y., et al. (2010). Sarcopenic obesity: prevalence and association with metabolic syndrome in the Korean Longitudinal Study on Health and Aging (KLoSHA). *Diabetes care* 33(7)**,** 1652-1654. doi: 10.2337/dc10-0107.

Newman, A., Kupelian, V., Visser, M., Simonsick, E., Goodpaster, B., Nevitt, M., et al. (2003). Sarcopenia: alternative definitions and associations with lower extremity function. *Journal of the American Geriatrics Society* 51(11)**,** 1602-1609. doi: 10.1046/j.1532-5415.2003.51534.x.

Park, J., Kwon, Y., and Park, H. (2017). Effects of 24-Week Aerobic and Resistance Training on Carotid Artery Intima-Media Thickness and Flow Velocity in Elderly Women with Sarcopenic Obesity. *J Atheroscler Thromb* 24(11)**,** 1117-1124. doi: 10.5551/jat.39065.

Studenski, S., Peters, K., Alley, D., Cawthon, P., McLean, R., Harris, T., et al. (2014). The FNIH sarcopenia project: rationale, study description, conference recommendations, and final estimates. *The journals of gerontology. Series A, Biological sciences and medical sciences* 69(5)**,** 547-558. doi: 10.1093/gerona/glu010.

Use, W.H.O.E.C.o.P.S.t., and Anthropometry, I.o. (1995). Physical status: the use and interpretation of anthropometry. Report of a WHO Expert Committee. *Geneva, Switzerland, WHO, 1995* 854(6)**,** 1-452. doi: 10.1002/(SICI)1520-6300(1996)8:6<786::AID-AJHB11>3.0.CO;2-I.

Vasconcelos, K.S.S., Dias, J.M.D., Araujo, M.C., Pinheiro, A.C., Moreira, B.S., and Dias, R.C. (2016). Effects of a progressive resistance exercise program with high-speed component on the physical function of older women with sarcopenic obesity: a randomized controlled trial. *Brazilian Journal Of Physical Therapy* 20(5)**,** 432-440. doi: 10.1590/bjpt-rbf.2014.0174.

Wang, L.Z., Guo, Y.B., and Lou, J.H. (2019). Effects of Home Exercise on Sarcopenia Obesity for Aging People. *Chin J Rehabil Theory Pract* 25(1)**,** 90-96. doi: 10.3969/j.issn.1006⁃9771.2019.01.012.
