# Supplementary material for: Effects of three modes of physical activity on physical fitness and hematological parameters in older people with sarcopenic obesity: A systematic review and meta-analysis
Source: Front Physiol. 2022 Aug 25;13:917525. doi: 10.3389/fphys.2022.917525 (PMC9458075; doi:10.3389/fphys.2022.917525)
Supplement: Supplementary file 2 [file Table2.DOCX]

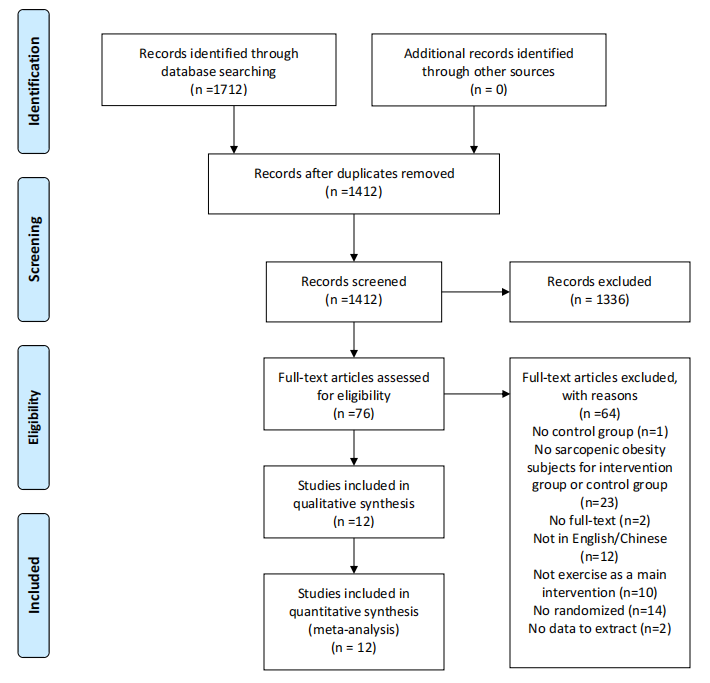


Fig.1 Flow of screening and selecting process according to Preferred Reporting Items for Systematic Reviews and meta-analysis (PRISMA).

A
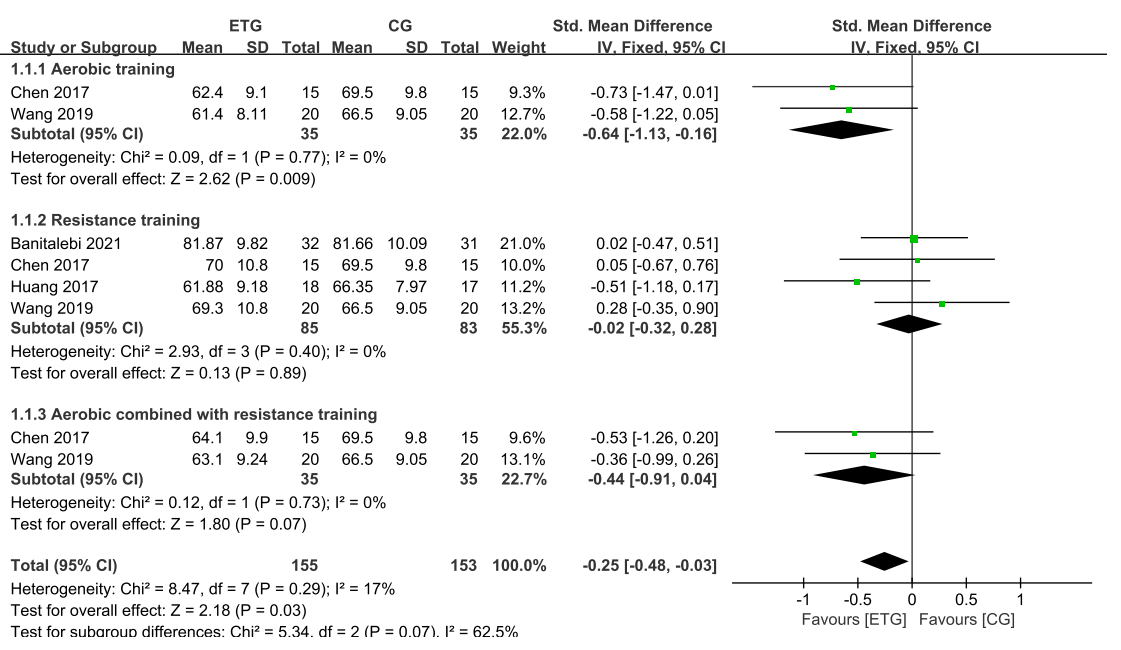


B


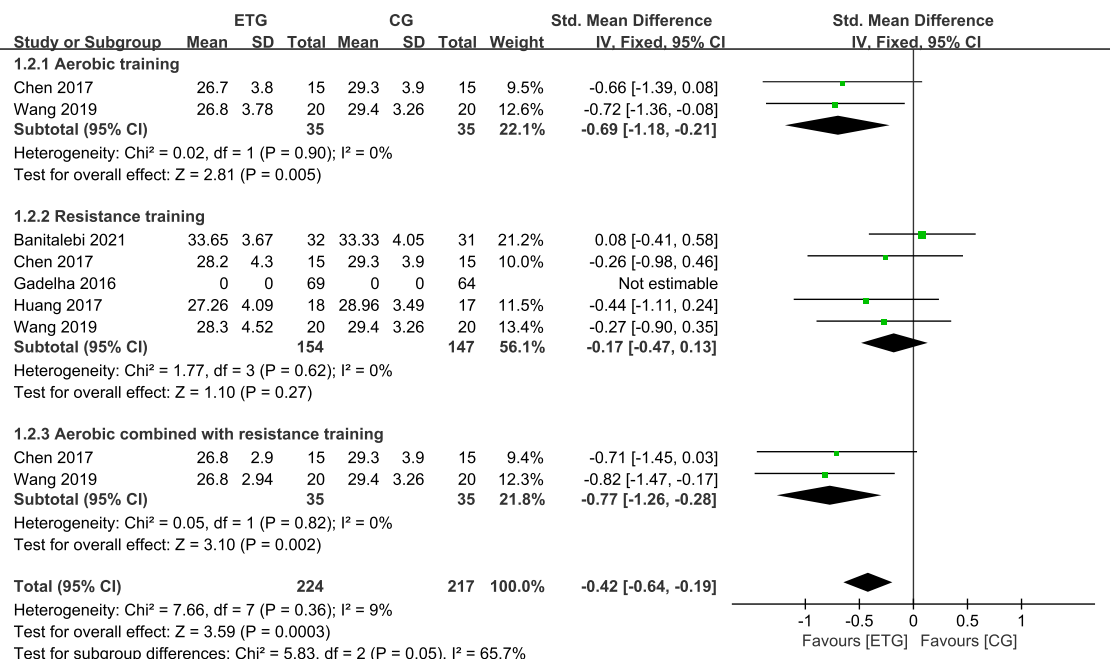


C


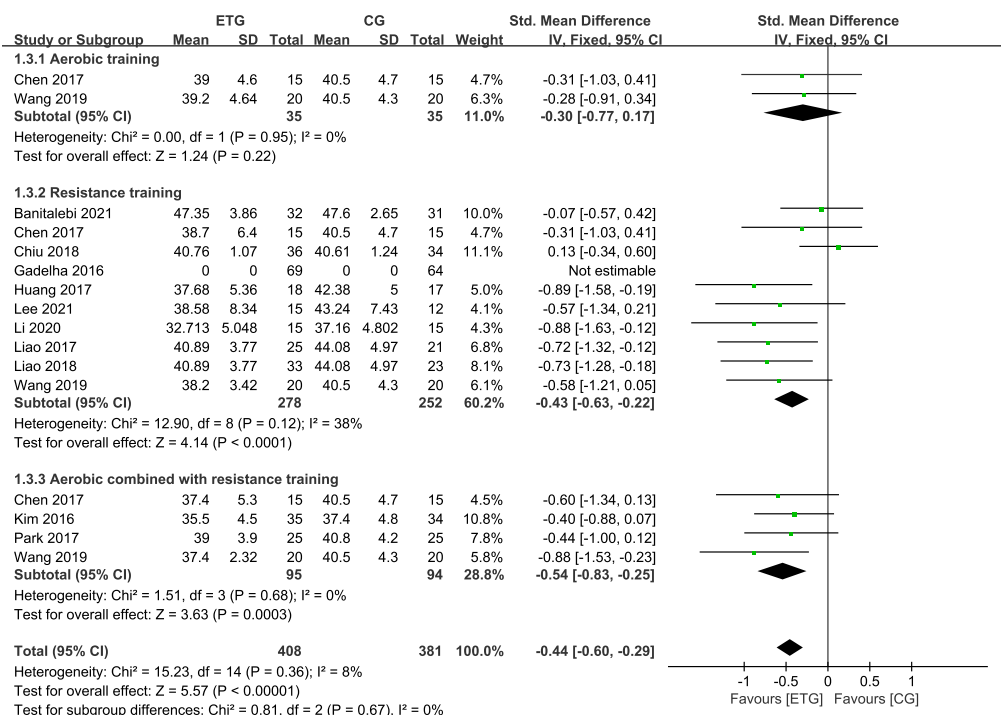


Fig. 2 Forest plots of the comparison of the exercise training group (ETG) versus the control group (CG) on a: body weight (BW); b: body mass index (BMI); c: percentage of body fat (BF%); CI: confidence interval; SD: standard deviation.

A
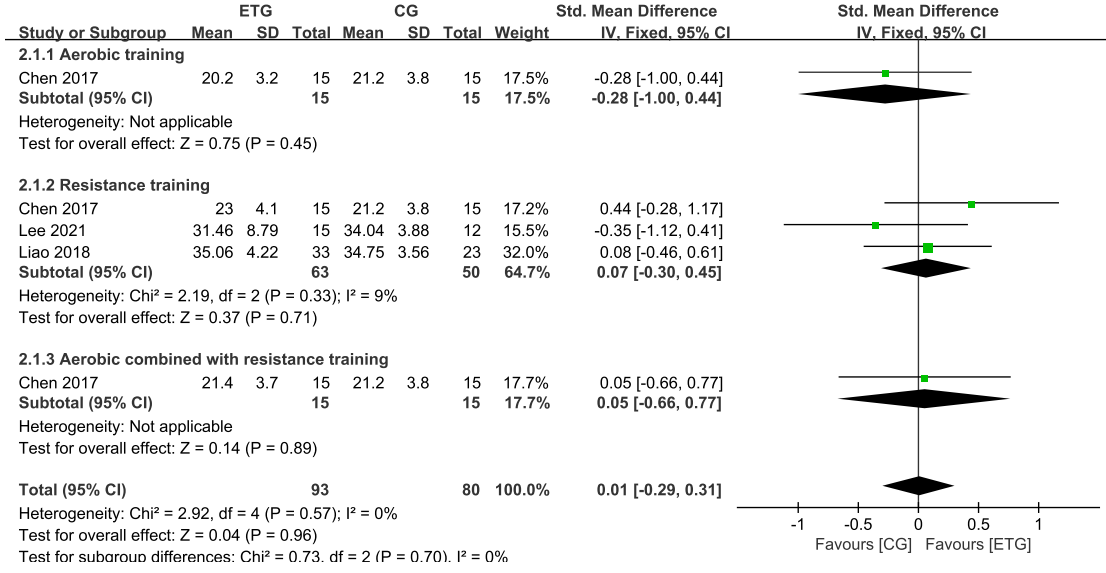


B
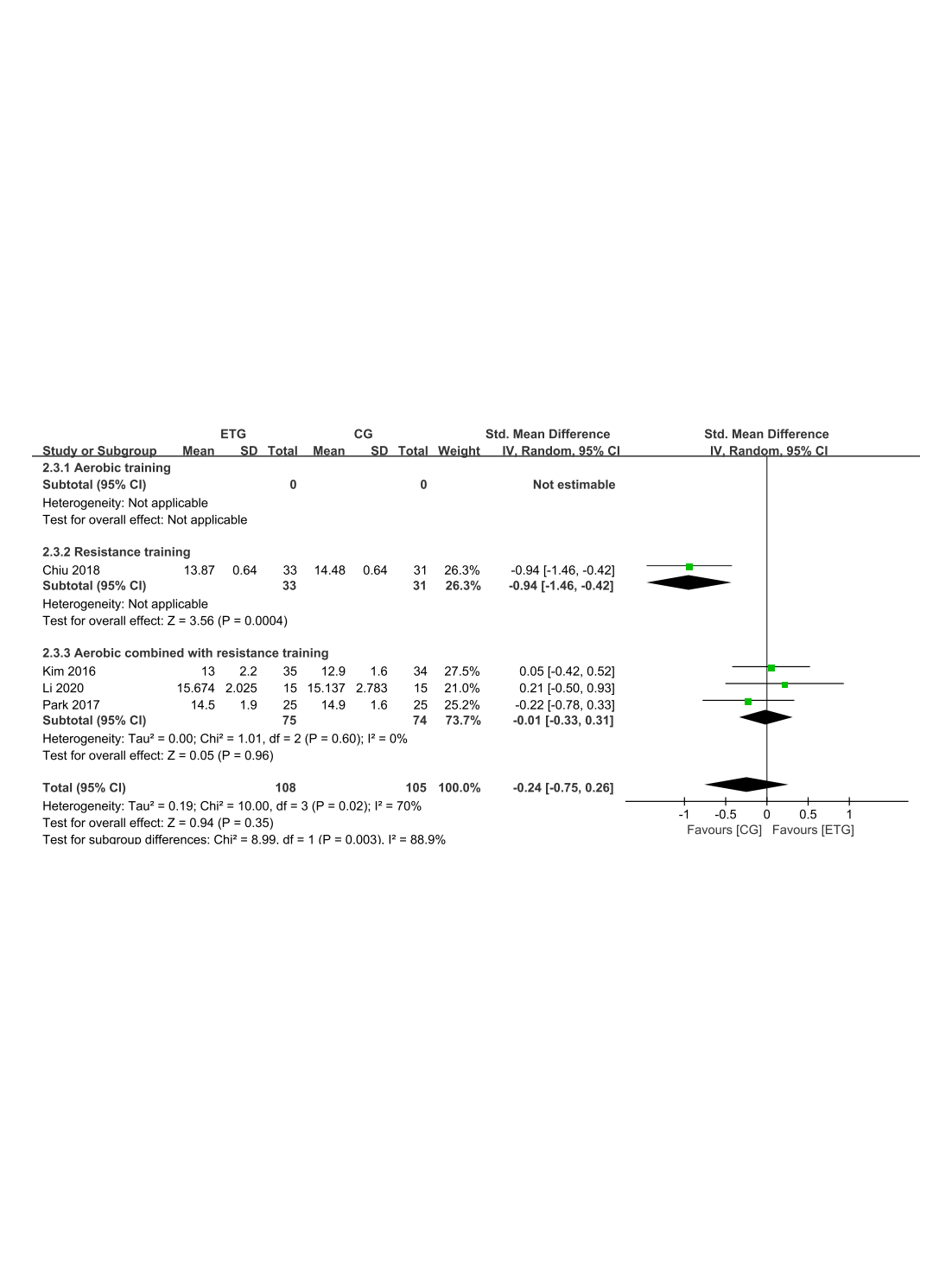


C
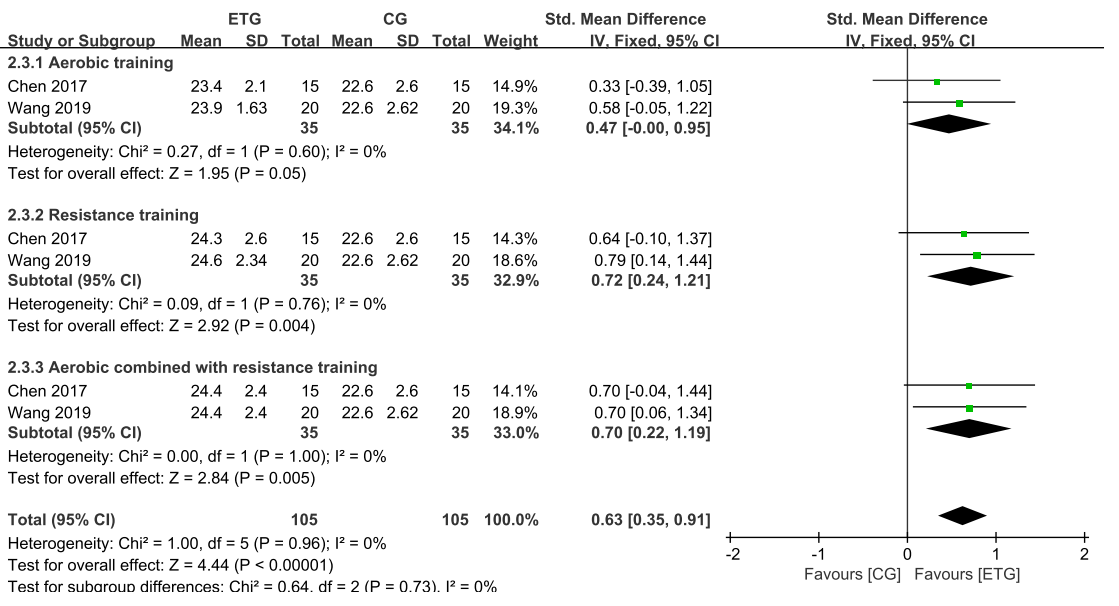


Fig. 3 Forest plots of the comparison of the exercise training group (ETG) versus the control group (CG) on a: skeletal muscle mass (SM); b: appendicular skeletal muscle mass (ASM); and c: appendicular skeletal muscle mass index (ASMI); CI: confidence interval; SD: standard deviation.

A
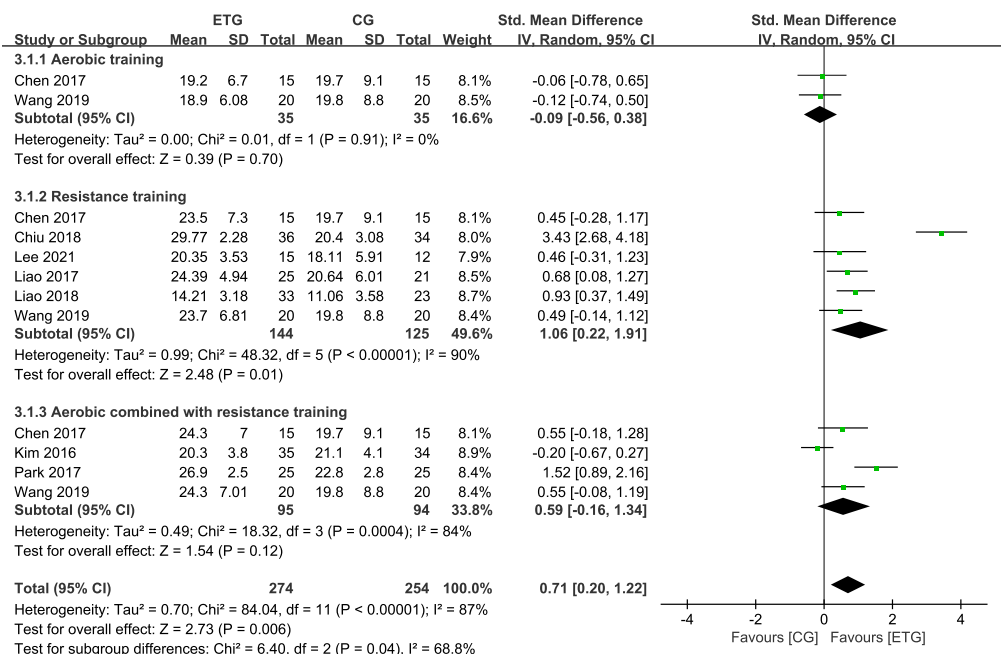


B


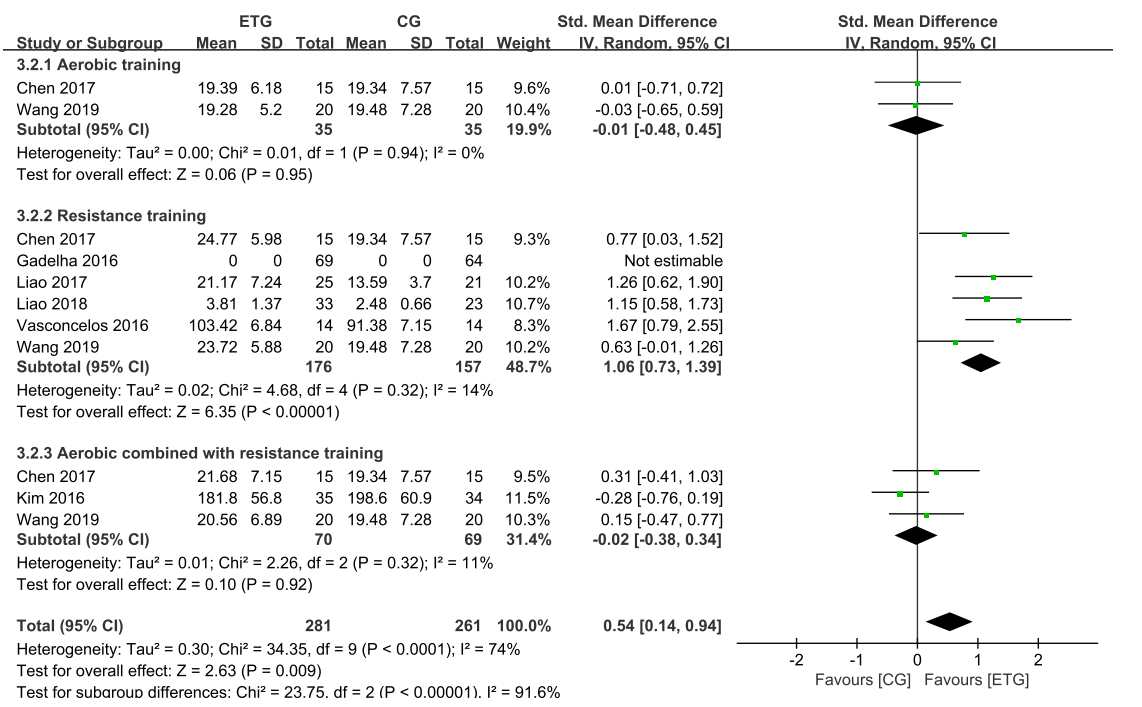


Fig.4 Forest plots of the comparison of the exercise training group (ETG) versus the control group (CG) on a: handgrip strength (HG); b: knee extension strength (KES); CI: confidence interval; SD: standard deviation.


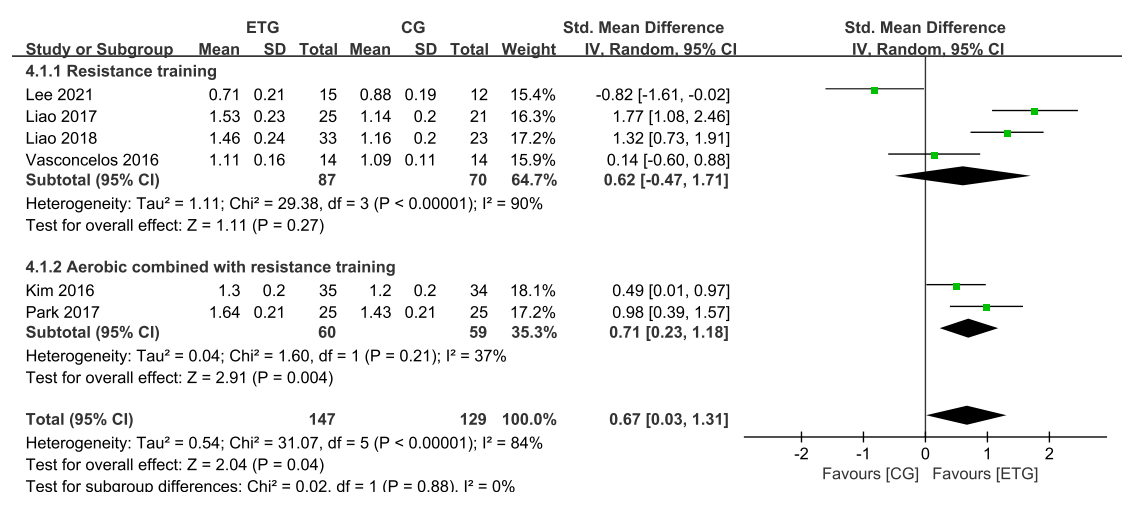


Fig.5 Forest plots of the comparison of the exercise training group (ETG) versus the control group (CG) on gait speed (GS); CI: confidence interval; SD: standard deviation.

A


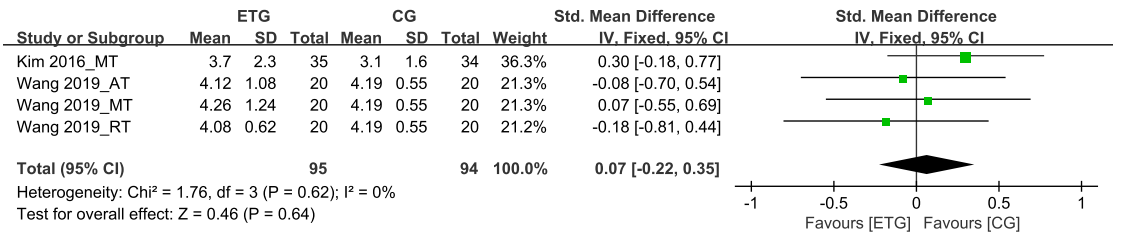


B


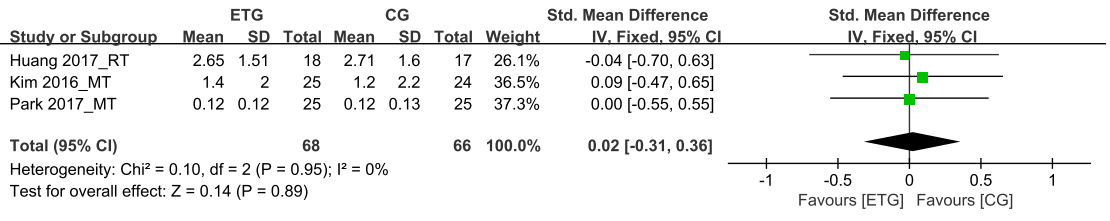


C
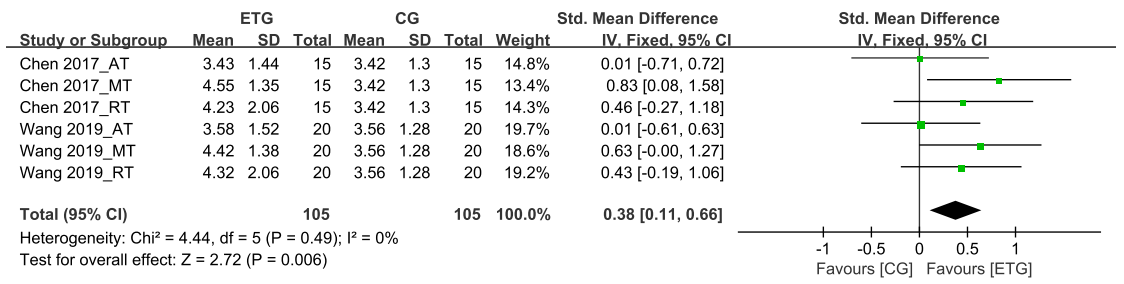


D
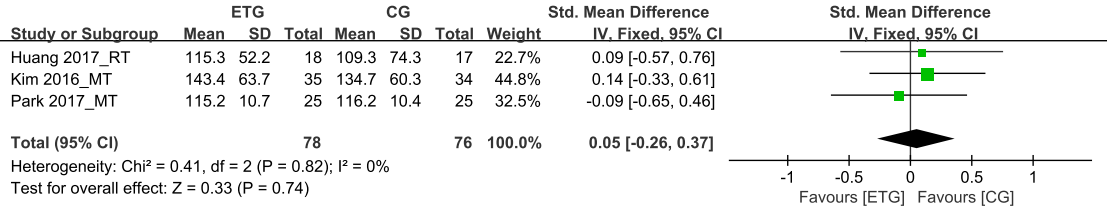


E
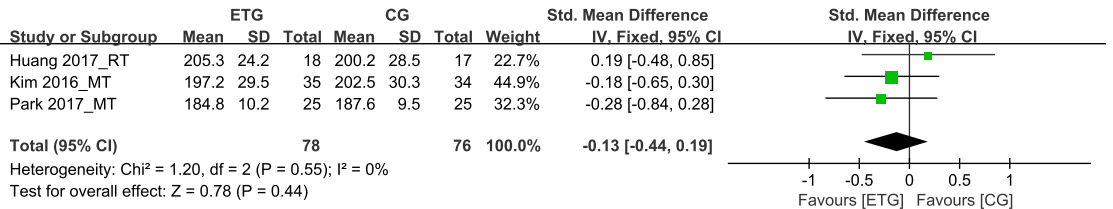


F
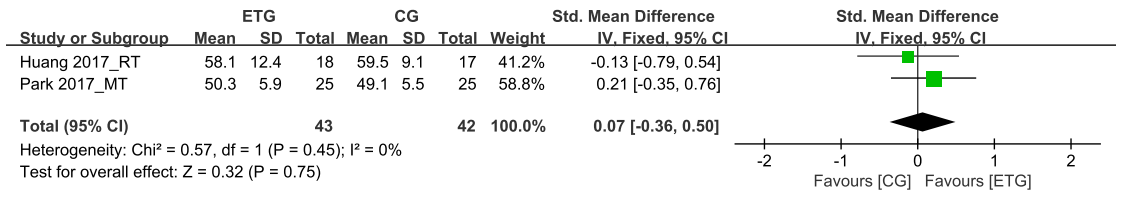


G
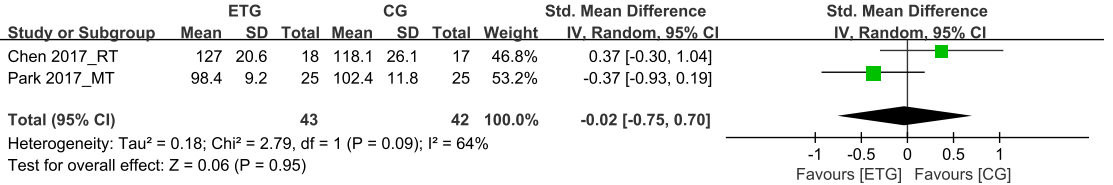


Fig.6 Forest plots of the comparison of the exercise training group (ETG) versus the control group (CG) on a: interleukin-6 (IL-6); b: C-reactive protein (CRP); c: insulin-like growth factor 1 (IGF-1); d: triglyceride (TG); e: total cholesterol (TC); f: high density lipoprotein (HDL); g: low density lipoprotein (LDL); AT: aerobic training; RT: resistance training; MT: aerobic combined with resistance training; CI: confidence interval; SD: standard deviation.
