# Supplementary material for: Effects of three modes of physical activity on physical fitness and hematological parameters in older people with sarcopenic obesity: A systematic review and meta-analysis
Source: Front Physiol. 2022 Aug 25;13:917525. doi: 10.3389/fphys.2022.917525 (PMC9458075; doi:10.3389/fphys.2022.917525)
Supplement: Supplementary file 3 [file Table3.DOCX]

Pubmed:

Search: (((((((((("Aging"[Mesh]) OR (((Senescence[Title/Abstract]) OR (Biological Aging[Title/Abstract])) OR (Biological Aging[Title/Abstract]))) OR ("Aged"[Mesh])) OR (Elderly[Title/Abstract])) OR ("Aged, 80 and over"[Mesh])) OR (((((((Oldest Old[Title/Abstract]) OR (Nonagenarians[Title/Abstract])) OR (Nonagenarian[Title/Abstract])) OR (Octogenarians[Title/Abstract])) OR (Octogenarian[Title/Abstract])) OR (Centenarians[Title/Abstract])) OR (Centenarian[Title/Abstract]))) OR ("Frail Elderly"[Mesh])) OR ((((((((((((((Elderly, Frail[Title/Abstract]) OR (Frail Elders[Title/Abstract])) OR (Elder, Frail[Title/Abstract])) OR (Elders, Frail[Title/Abstract])) OR (Frail Elder[Title/Abstract])) OR (Functionally-Impaired Elderly[Title/Abstract])) OR (Elderly, Functionally-Impaired[Title/Abstract])) OR (Functionally Impaired Elderly[Title/Abstract])) OR (Frail Older Adults[Title/Abstract])) OR (Adult, Frail Older[Title/Abstract])) OR (Adults, Frail Older[Title/Abstract])) OR (Frail Older Adult[Title/Abstract])) OR (Older Adult, Frail[Title/Abstract])) OR (Older Adults, Frail[Title/Abstract]))) AND ((((((("Sarcopenia"[Mesh]) OR (Sarcopenias[Title/Abstract])) OR (Sarcopenic[Title/Abstract])) OR (Muscle loss[Title/Abstract])) OR (Muscle wasting[Title/Abstract])) OR (((((((((((((((((((("Muscular Atrophy"[Mesh]) OR (Atrophies, Muscular[Title/Abstract])) OR (Atrophy, Muscular[Title/Abstract])) OR (Muscular Atrophies[Title/Abstract])) OR (Atrophy, Muscle[Title/Abstract])) OR (Atrophies, Muscle[Title/Abstract])) OR (Muscle Atrophies[Title/Abstract])) OR (Muscle Atrophy[Title/Abstract])) OR (Neurogenic Muscular Atrophy[Title/Abstract])) OR (Atrophies, Neurogenic Muscular[Title/Abstract])) OR (Atrophy, Neurogenic Muscular[Title/Abstract])) OR (Muscular Atrophies, Neurogenic[Title/Abstract])) OR (Muscular Atrophy, Neurogenic[Title/Abstract])) OR (Neurogenic Muscular Atrophies[Title/Abstract])) OR (Neurotrophic Muscular Atrophy[Title/Abstract])) OR (Atrophies, Neurotrophic Muscular[Title/Abstract])) OR (Atrophy, Neurotrophic Muscular[Title/Abstract])) OR (Muscular Atrophies, Neurotrophic[Title/Abstract])) OR (Muscular Atrophy, Neurotrophic[Title/Abstract])) OR (Neurotrophic Muscular Atrophies[Title/Abstract]))) OR (((((Age related muscle loss[Title/Abstract]) OR (Age-related muscle loss[Title/Abstract])) OR (Muscle insufficiency[Title/Abstract])) OR (Muscle depletion[Title/Abstract])) OR (Skeletal muscle depletion[Title/Abstract])))) AND ((("Obesity"[Mesh]) OR (Obese[Title/Abstract])) OR (Overweight[Title/Abstract]))) AND (((((((((((((((((((((((((((((((("Exercise"[Mesh]) OR (Exercises[Title/Abstract])) OR (Physical Activity[Title/Abstract])) OR (Activities, Physical[Title/Abstract])) OR (Activity, Physical[Title/Abstract])) OR (Physical Activities[Title/Abstract])) OR (Exercise, Physical[Title/Abstract])) OR (Exercises, Physical[Title/Abstract])) OR (Physical Exercise[Title/Abstract])) OR (Physical Exercises[Title/Abstract])) OR (Acute Exercise[Title/Abstract])) OR (Acute Exercises[Title/Abstract])) OR (Exercise, Acute[Title/Abstract])) OR (Exercises, Acute[Title/Abstract])) OR (Exercise, Isometric[Title/Abstract])) OR (Exercises, Isometric[Title/Abstract])) OR (Isometric Exercises[Title/Abstract])) OR (Isometric Exercise[Title/Abstract])) OR (Exercise, Aerobic[Title/Abstract])) OR (Aerobic Exercise[Title/Abstract])) OR (Aerobic Exercises[Title/Abstract])) OR (Exercises, Aerobic[Title/Abstract])) OR (Exercise Training[Title/Abstract])) OR (Exercise Trainings[Title/Abstract])) OR (Training, Exercise[Title/Abstract])) OR (Traings, Exercise[Title/Abstract])) OR (((("Motor Activity"[Mesh]) OR (Activities, Motor[Title/Abstract])) OR (Activity, Motor[Title/Abstract])) OR (Motor Activities[Title/Abstract]))) OR (("Movement"[Mesh]) OR (Movements[Title/Abstract]))) OR (((((((((((("Exercise Therapy"[Mesh]) OR (Remedial Exercise[Title/Abstract])) OR (Exercise, Remedial[Title/Abstract])) OR (Exercises, Remedial[Title/Abstract])) OR (Remedial Exercises[Title/Abstract])) OR (Therapy, Exercise[Title/Abstract])) OR (Exercise Therapies[Title/Abstract])) OR (Therapies, Exercise[Title/Abstract])) OR (Rehabilitation Exercise[Title/Abstract])) OR (Exercise, Rehabilitation[Title/Abstract])) OR (Exercises, Rehabilitation[Title/Abstract])) OR (Rehabilitation Exercises[Title/Abstract]))) OR ((Training[Title/Abstract]) OR (physical therapy[Title/Abstract]))) OR (("Endurance Training"[Mesh]) OR (Training, Endurance[Title/Abstract]))) OR (((((((((((((((((((((((("Resistance Training"[Mesh]) OR (Training, Resistance[Title/Abstract])) OR (Strength Training[Title/Abstract])) OR (Training, Strength[Title/Abstract])) OR (Weight-Lifting Strengthening Program[Title/Abstract])) OR (Strengthening Program, Weight-Lifting[Title/Abstract])) OR (Strengthening Programs, Weight-Lifting[Title/Abstract])) OR (Weight Lifting Strengthening Program[Title/Abstract])) OR (Weight-Lifting Strengthening Programs[Title/Abstract])) OR (Weight-Lifting Exercise Program[Title/Abstract])) OR (Exercise Program, Weight-Lifting[Title/Abstract])) OR (Exercise Programs, Weight-Lifting[Title/Abstract])) OR (Weight Lifting Exercise Program[Title/Abstract])) OR (Weight-Lifting Exercise Programs[Title/Abstract])) OR (Weight-Bearing Strengthening Program[Title/Abstract])) OR (Strengthening Program, Weight-Bearing[Title/Abstract])) OR (Strengthening Programs, Weight-Bearing[Title/Abstract])) OR (Weight Bearing Strengthening Program[Title/Abstract])) OR (Weight-Bearing Strengthening Programs[Title/Abstract])) OR (Weight-Bearing Exercise Program[Title/Abstract])) OR (Exercise Program, Weight-Bearing[Title/Abstract])) OR (Exercise Programs, Weight-Bearing[Title/Abstract])) OR (Weight Bearing Exercise Program[Title/Abstract])) OR (Weight-Bearing Exercise Programs[Title/Abstract]))) Filters: from 2010 - 2021

Cochrane library:

#1 MeSH descriptor: [Aging] explode all trees

#2 (Biological Aging OR Aging, Biological OR Senescence):ti,ab,kw (Word variations have been searched)

#3 MeSH descriptor: [Aged] explode all trees

#4 (Elderly):ti,ab,kw (Word variations have been searched)

#5 MeSH descriptor: [Cognitive Aging] explode all trees

#6 (Aging, Cognitive):ti,ab,kw (Word variations have been searched)

#7 MeSH descriptor: [Aged, 80 and over] explode all trees

#8 (Nonagenarians OR Nonagenarian OR Oldest Old OR Centenarians OR Centenarian OR Octogenarian OR Octogenarians):ti,ab,kw (Word variations have been searched)

#9 MeSH descriptor: [Frail Elderly] explode all trees

#10 (Elderly, Functionally-Impaired OR Functionally-Impaired Elderly OR Functionally Impaired Elderly OR Adults, Frail Older OR Older Adult, Frail OR Frail Older Adults OR Frail Older Adult OR Adult, Frail Older OR Older Adults, Frail OR Frail Elders OR Elders, Frail OR Frail Elder OR Elderly, Frail OR Elder, Frail):ti,ab,kw (Word variations have been searched)

#11 #1 OR #2 OR #3 OR #4 OR #5 OR #6 OR #7 OR #8 OR #9 OR #10

#12 MeSH descriptor: [Sarcopenia] explode all trees

#13 (Sarcopenias):ti,ab,kw (Word variations have been searched)

#14 (Sarcopenic):ti,ab,kw (Word variations have been searched)

#15 (Muscle loss):ti,ab,kw (Word variations have been searched)

#16 (Muscle wasting):ti,ab,kw (Word variations have been searched)

#17 MeSH descriptor: [Muscular Atrophy] explode all trees

#18 (Neurogenic Muscular Atrophy OR Atrophy, Neurogenic Muscular OR Atrophies, Neurotrophic Muscular OR Neurogenic Muscular Atrophies OR Neurotrophic Muscular Atrophy OR Muscular Atrophies, Neurotrophic OR Neurotrophic Muscular Atrophies OR Muscular Atrophy, Neurogenic OR Atrophies, Neurogenic Muscular OR Atrophy, Neurotrophic Muscular OR Muscular Atrophies, Neurogenic OR Muscular Atrophy, Neurotrophic OR Atrophies, Muscle OR Muscle Atrophy OR Muscle Atrophies OR Atrophy, Muscular OR Atrophies, Muscular OR Muscular Atrophies OR Atrophy, Muscle):ti,ab,kw (Word variations have been searched)

#19 (Age related muscle loss):ti,ab,kw (Word variations have been searched)

#20 (Age-related muscle loss):ti,ab,kw (Word variations have been searched)

#21 (Muscle insufficiency):ti,ab,kw (Word variations have been searched)

#22 (Muscle depletion):ti,ab,kw (Word variations have been searched)

#23 (Skeletal muscle depletion):ti,ab,kw (Word variations have been searched)

#24 #12 OR #13 OR #14 OR #15 OR #16 OR #17 OR #18 OR #19 OR #20 OR #21 OR #22 OR #23

#25 MeSH descriptor: [Obesity] explode all trees

#26 (Obese):ti,ab,kw (Word variations have been searched)

#27 (sarcopenic obesity):ti,ab,kw (Word variations have been searched)

#28 #25 OR #26 OR #27

#29 MeSH descriptor: [Exercise] explode all trees

#30 (Exercises, Isometric OR Exercise, Isometric OR Isometric Exercises OR Isometric Exercise OR Acute Exercise OR Acute Exercises OR Exercises, Acute OR Exercise, Acute OR Exercise, Aerobic OR Aerobic Exercise OR Exercises, Aerobic OR Aerobic Exercises OR Activities, Physical OR Physical Exercises OR Exercises OR Physical Activities OR Activity, Physical OR Exercises, Physical OR Physical Exercise OR Physical Activity OR Exercise, Physical OR Trainings, Exercise OR Exercise Trainings OR Training, Exercise OR Exercise Training):ti,ab,kw (Word variations have been searched)

#31 MeSH descriptor: [Motor Activity] explode all trees

#32 (Activity, Motor OR Activities, Motor OR Motor Activities):ti,ab,kw (Word variations have been searched)

#33 (Movement (physiology)):ti,ab,kw (Word variations have been searched)

#34 MeSH descriptor: [Movement] explode all trees

#35 (Movements):ti,ab,kw (Word variations have been searched)

#36 (kinesiotherapy):ti,ab,kw (Word variations have been searched)

#37 (Training):ti,ab,kw (Word variations have been searched)

#38 MeSH descriptor: [Physical Therapy Modalities] explode all trees

#39 (Therapy, Physical OR Physical Therapy OR Physical Therapies OR Physiotherapies (Techniques) OR Physical Therapy Technique OR Modalities, Physical Therapy OR Physiotherapy (Techniques) OR Modality, Physical Therapy OR Techniques, Physical Therapy OR Physical Therapy Techniques OR Physical Therapy Modality OR Physiotherapy, Group OR Physiotherapies, Group OR Group Physiotherapies OR Group Physiotherapy OR Neurophysiotherapy OR Neurological Physiotherapy OR Physiotherapy, Neurological):ti,ab,kw (Word variations have been searched)

#40 MeSH descriptor: [Endurance Training] explode all trees

#41 (Training, Endurance):ti,ab,kw (Word variations have been searched)

#42 MeSH descriptor: [Resistance Training] explode all trees

#43 (Weight-Lifting Strengthening Program OR Weight-Lifting Exercise Program OR Weight-Lifting Strengthening Programs OR Strengthening Program, Weight-Lifting OR Weight Lifting Strengthening Program OR Weight Lifting Exercise Program OR Exercise Programs, Weight-Lifting OR Weight-Lifting Exercise Programs OR Exercise Program, Weight-Lifting OR Strengthening Programs, Weight-Lifting OR Training, Strength OR Strength Training OR Training, Resistance OR Exercise Programs, Weight-Bearing OR Strengthening Programs, Weight-Bearing OR Weight-Bearing Strengthening Programs OR Weight Bearing Strengthening Program OR Exercise Program, Weight-Bearing OR Weight-Bearing Strengthening Program OR Weight-Bearing Exercise Programs OR Strengthening Program, Weight-Bearing OR Weight-Bearing Exercise Program OR Weight Bearing Exercise Program):ti,ab,kw (Word variations have been searched)

#44 #29 OR #30 OR #31 OR #32 OR #33 OR #34 OR #35 OR #36 OR #37 OR #38 OR #39 OR #40 OR #41 OR #42 OR #43

#45 #11 AND #24 AND #28 AND #44

Embase:

#50. #49 AND (2010:py OR 2011:py OR 2012:py OR 2013:py OR 2014:py OR 2015:py OR 2016:py OR 2017:py OR 2018:py OR 2019:py OR 2020:py OR 2021:py)

#49. #8 AND #16 AND #22 AND #48

#48. #25 OR #28 OR #31 OR #32 OR #35 OR #38 OR #41 OR #44 OR #47

#47. #45 OR #46

#46. 'resistance exercise':ab,ti OR 'resistance exercise training':ab,ti OR 'resistance-type exercise':ab,ti OR 'resistance-type training':ab,ti OR 'strength training':ab,ti OR 'strength-type exercise':ab,ti OR 'strength-type training':ab,ti

#45. 'resistance training'/exp

#44. #42 OR #43

#43. 'endurance exercise':ab,ti OR 'endurance exercise training':ab,ti OR 'endurance workout':ab,ti OR 'endurance-type exercise':ab,ti OR 'endurance-type training':ab,ti

#42. 'endurance training'/exp

#41. #39 OR #40

#40. 'physical therapy':ab,ti OR 'physical therapy (speciality)':ab,ti OR 'physical therapy (specialty)':ab,ti OR 'physical therapy modalities':ab,ti OR 'physical therapy service':ab,ti OR 'physical therapy speciality':ab,ti OR 'physical therapy specialty':ab,ti OR 'physical therapy techniques':ab,ti OR 'physical treatment':ab,ti OR 'physio therapy':ab,ti OR 'physiotherapy department':ab,ti OR 'therapy, physical':ab,ti

#39. 'physiotherapy'/exp

#38. #36 OR #37

#37. 'army training':ab,ti OR 'athletic training':ab,ti OR 'athletic training program':ab,ti OR 'athletic training programme':ab,ti OR detraining:ab,ti OR 'military training':ab,ti OR 'physical training':ab,ti OR 'sport specific training':ab,ti OR 'technical training':ab,ti OR 'training athlete':ab,ti OR 'training course':ab,ti OR 'training program':ab,ti OR 'training programme':ab,ti OR 'training, athletic':ab,ti OR 'training, military':ab,ti OR 'training, physical':ab,ti

#36. 'training'/exp

#35. #33 OR #34

#34. 'corrective exercise':ab,ti OR 'exercise movement techniques':ab,ti OR 'exercise therapy':ab,ti OR 'exercise treatment':ab,ti OR 'kinesiotherapeutic intervention':ab,ti OR 'kinesiotherapeutic method':ab,ti OR 'kinesiotherapeutic procedure':ab,ti OR 'kinesiotherapeutic technique':ab,ti OR 'kinesiotherapeutical treatment':ab,ti OR 'kinesitherapeutic exercises':ab,ti OR 'kinesitherapeutic intervention':ab,ti OR 'kinesitherapeutic method':ab,ti OR 'kinesitherapeutic methodology':ab,ti OR 'kinesitherapeutic procedure':ab,ti OR 'kinesitherapeutic technique':ab,ti OR 'kinesitherapeutic treatment':ab,ti OR 'kinesitherapeutical treatment':ab,ti OR kinesitherapy:ab,ti OR 'sktm (specialized kinesitherapeutic methodology)':ab,ti OR 'specialised kinesitherapeutic methodology':ab,ti OR 'specialized kinesitherapeutic methodology':ab,ti OR 'therapeutic exercise':ab,ti OR 'therapy, exercise':ab,ti OR 'treatment, exercise':ab,ti

#33. 'kinesiotherapy'/exp

#32. movements:ab,ti

#31. #29 OR #30

#30. movement:ab,ti

#29. 'movement (physiology)'/exp

#28. #26 OR #27

#27. 'activity, motor':ab,ti

#26. 'motor activity'/exp

#25. #23 OR #24

#24. 'biometric exercise':ab,ti OR effort:ab,ti OR 'exercise capacity':ab,ti OR 'exercise performance':ab,ti OR 'exercise training':ab,ti OR exertion:ab,ti OR 'fitness training':ab,ti OR 'fitness workout':ab,ti OR 'physical conditioning, human':ab,ti OR 'physical effort':ab,ti OR 'physical exercise':ab,ti OR 'physical exertion':ab,ti OR 'physical work-out':ab,ti OR 'physical workout':ab,ti

#23. 'exercise'/exp

#22. #19 OR #20 OR #21

#21. 'sarcopenic obesity'/exp

#20. obese:ab,ti

#19. #17 OR #18

#18. 'adipose tissue hyperplasia':ab,ti OR 'adipositas':ab,ti OR adiposity:ab,ti OR 'alimentary obesity':ab,ti OR 'body weight, excess':ab,ti OR 'corpulency':ab,ti OR 'fat overload syndrome':ab,ti OR 'nutritional obesity':ab,ti OR obesitas:ab,ti OR overweight:ab,ti

#17. 'obesity'/exp

#16. #11 OR #14 OR #15

#15. 'age related muscle loss':ab,ti OR 'age-related muscle loss':ab,ti OR 'muscle insufficiency':ab,ti OR 'muscle depletion':ab,ti OR 'skeletal muscle depletion':ab,ti

#14. #12 OR #13

#13. amyotrophia:ab,ti OR amyotrophy:ab,ti OR 'atrophic muscular disorders':ab,ti OR 'atrophy type 2':ab,ti OR 'atrophy, muscle':ab,ti OR 'degeneration, muscle':ab,ti OR 'hirayama disease':ab,ti OR 'muscle atrophia':ab,ti OR 'muscle cell degeneration':ab,ti OR 'muscle degeneration':ab,ti OR 'muscle fiber atrophy':ab,ti OR 'muscle fiber degeneration':ab,ti OR 'muscle recession':ab,ti OR 'muscle wasting':ab,ti OR 'muscular atrophy':ab,ti OR 'muscular degeneration':ab,ti OR 'muscular disorders, atrophic':ab,ti OR 'myoatrophy':ab,ti OR 'myodegeneration':ab,ti OR 'myofibrillar degeneration':ab,ti OR 'myophagism':ab,ti

#12. 'muscle atrophy'/exp

#11. #9 OR #10

#10. sarcopenias:ab,ti OR sarcopenic:ab,ti OR 'muscle loss':ab,ti OR 'muscle wasting':ab,ti

#9. 'sarcopenia'/exp

#8. #3 OR #6 OR #7

#7. 'aged, 80 and over':ab,ti OR 'frail elderly':ab,ti

#6. #4 OR #5

#5. 'aged patient':ab,ti OR 'aged people':ab,ti OR 'aged person':ab,ti OR 'aged subject':ab,ti OR elderly:ab,ti OR 'elderly patient':ab,ti OR 'elderly people':ab,ti OR 'elderly person':ab,ti OR 'elderly subject':ab,ti OR 'senior citizen':ab,ti OR senium:ab,ti

#4. 'aged'/exp

#3. #1 OR #2

#2. 'age induced changes':ab,ti OR ageing:ab,ti OR 'aging biology':ab,ti OR 'aging process':ab,ti OR 'aging rate':ab,ti

#1. 'aging'/exp

Web of Science:

#1

AB=(Aging OR Senescence OR Biological Aging OR Aging, Biological OR Aged OR Elderly OR Aged, 80 and over OR Oldest Old OR Nonagenarians OR Nonagenarian OR Octogenarians OR Octogenarian OR Centenarians OR Centenarian OR Frail Elderly OR Elderly, Frail OR Frail Elders OR Elder, Frail OR Elders, Frail OR Frail Elder OR Functionally-Impaired Elderly OR Elderly, Functionally-Impaired OR Functionally Impaired Elderly OR Frail Older Adults OR Adult, Frail Older OR Adults, Frail Older OR Frail Older Adult OR Older Adult, Frail OR Older Adults, Frail )

#2

AB=(Sarcopenia OR Sarcopenias OR Sarcopenic OR Muscle loss OR Muscle wasting OR Muscular Atrophy OR Atrophies, Muscular OR Atrophy, Muscular OR Muscular Atrophies OR Atrophy, Muscle OR Atrophies, Muscle OR Muscle Atrophies OR Muscle Atrophy OR Neurogenic Muscular Atrophy OR Atrophies, Neurogenic Muscular OR Atrophy, Neurogenic Muscular OR Muscular Atrophies, Neurogenic OR Muscular Atrophy, Neurogenic OR Neurogenic Muscular Atrophies OR Neurotrophic Muscular Atrophy OR Atrophies, Neurotrophic Muscular OR Atrophy, Neurotrophic Muscular OR Muscular Atrophies, Neurotrophic OR Muscular Atrophy, Neurotrophic OR Neurotrophic Muscular Atrophies OR Age related muscle loss OR Age-related muscle loss OR Muscle insufficiency OR Muscle depletion OR Skeletal muscle depletion )

#3

AB=(Obesity OR Obese OR Overweight)

#4

AB=(exercise OR Exercises OR Physical Activity OR Activities, Physical OR Activity, Physical OR Physical Activities OR Exercise, Physical OR Exercises, Physical OR Physical Exercise OR Physical Exercises OR Acute Exercise OR Acute Exercises OR Exercise, Acute OR Exercises, Acute OR Exercise, Isometric OR Exercises, Isometric OR Isometric Exercises OR Isometric Exercise OR Exercise, Aerobic OR Aerobic Exercise OR Aerobic Exercises OR Exercises, Aerobic OR Exercise Training OR Exercise Trainings OR Training, Exercise OR Traings, Exercise )

#5

AB=(Motor Activity OR Activities, Motor OR Activity, Motor OR Motor Activities OR Movement OR Movements OR Exercise Therapy OR Remedial Exercise OR Exercise, Remedial OR Exercises, Remedial OR Remedial Exercises OR Therapy, Exercise OR Exercise Therapies OR Therapies, Exercise OR Rehabilitation Exercise OR Exercise, Rehabilitation OR Exercises, Rehabilitation OR Rehabilitation Exercises OR Training OR physical therapy OR Endurance Training OR Training, Endurance)

#6

AB=(Resistance Training OR Training, Resistance OR Strength Training OR Training, Strength OR Weight-Lifting Strengthening Program OR Strengthening Program, Weight-Lifting OR Strengthening Programs, Weight-Lifting OR Weight Lifting Strengthening Program OR Weight-Lifting Strengthening Programs OR Weight-Lifting Exercise Program OR Exercise Program, Weight-Lifting OR Exercise Programs, Weight-Lifting OR Weight Lifting Exercise Program OR Weight-Lifting Exercise Programs OR Weight-Bearing Strengthening Program OR Strengthening Program, Weight-Bearing OR Strengthening Programs, Weight-Bearing OR Weight Bearing Strengthening Program OR Weight-Bearing Strengthening Programs OR Weight-Bearing Exercise Program OR Exercise Program, Weight-Bearing OR Exercise Programs, Weight-Bearing OR Weight Bearing Exercise Program OR Weight-Bearing Exercise Programs)

#7

((#4) OR #5) OR #6

#8

(((#1) AND #2) AND #3) AND #7

#9

(((#1) AND #2) AND #3) AND #7 and 2010 or 2011 or 2012 or 2013 or 2014 or 2015 or 2016 or 2017 or 2018 or 2019 or 2020 or 2021 or 2022 (Publication Years)

#10

(((#1) AND #2) AND #3) AND #7 and 2010 or 2011 or 2012 or 2013 or 2014 or 2015 or 2016 or 2017 or 2018 or 2019 or 2020 or 2021 or 2022 (Publication Years) and 2022 (Publication Years)

#11

(((#1) AND #2) AND #3) AND #7 and 2010 or 2011 or 2012 or 2013 or 2014 or 2015 or 2016 or 2017 or 2018 or 2019 or 2020 or 2021 or 2022 (Publication Years)

#12

(((#1) AND #2) AND #3) AND #7 and 2022 or 2021 or 2020 or 2019 or 2018 or 2017 or 2016 or 2015 or 2014 or 2013 or 2012 or 2011 or 2010 (Publication Years)

The China National Knowledge Infrastructure (CNKI)：

（摘要 老年 或含 老人）并且（摘要 肌少症 或含 肌肉萎缩）并且（摘要 肥胖 或含 超重）并且（摘要 运动 或含 训练）

发表时间：从2010-01-01到2021-11-19

（摘要 老年 或含 老人）并且（摘要 少肌性 或含 肌少性）并且（摘要 肥胖 或含 超重）并且（摘要 运动 或含 训练）

发表时间：从2010-01-01到2021-11-21

（摘要 老年 或含 老人）并且（摘要 肌少症 或含 肌肉萎缩）并且（摘要 肥胖 或含 超重）并且（摘要 物理治疗 或含 练习）

发表时间：从2010-01-01到2021-11-19

Wanfang Data：

(摘要:(老年 OR 老人) and 摘要:(肌少症 OR 少肌性 OR 肌少性) and 摘要:(肥胖 OR 超重) and 摘要:(运动 OR 训练 OR 物理治疗 OR 练习)) and Date:2010-*
